# The circadian clock in immune cells controls the magnitude of *Leishmania* parasite infection

**DOI:** 10.1038/s41598-017-11297-8

**Published:** 2017-09-07

**Authors:** Silke Kiessling, Geneviève Dubeau-Laramée, Hyejee Ohm, Nathalie Labrecque, Martin Olivier, Nicolas Cermakian

**Affiliations:** 1 Douglas Mental Health University Institute, McGill University, Montreal, Quebec, Canada; 2 Maisonneuve-Rosemont Hospital Research Centre, Department of Medicine and Department of Microbiology, Infectiology and Immunology, University of Montreal, Montreal, Quebec, Canada; 3 Department of Medicine, Microbiology and Immunology, McGill University, and Infectious Diseases and Immunity in Global Health Program, Research Institute of the McGill University Health Centre, Montréal, Quebec, Canada; 4 Present Address: Chair of Nutrition and Immunology, Technical University of Munich, Freising, Germany

## Abstract

The intracellular parasite *Leishmania* uses neutrophils and macrophages as host cells upon infection. These immune cells harbour their own intrinsic circadian clocks, known to influence many aspects of their functions. Therefore, we tested whether the host circadian clocks regulate the magnitude of *Leishmania major* infection in mice. The extent of parasitic infection varied over 24 h in bone marrow-derived macrophages *in vitro* and in two different *in vivo* models, footpad and peritoneal cavity infection. *In vivo* this was paralleled by time of day-dependent neutrophil and macrophage infiltration to the infection site and rhythmic chemokine expression. Thus, rhythmic parasitic infection observed *in vivo* was likely initiated by the circadian expression of chemoattractants and the subsequent rhythmic infiltration of neutrophils and macrophages. Importantly, all rhythms were abolished in clock-deficient macrophages and when mice lacking the circadian clock in immune cells were infected. Therefore we demonstrated a critical role for the circadian clocks in immune cells in modulating the magnitude of *Leishmania* infection. To our knowledge this is the first report showing that the circadian clock controls infection by protozoan parasites in mammals. Understanding the timed regulation of host-parasite interactions will allow developing better prophylactic and therapeutic strategies to fight off vector-borne diseases.

## Introduction

Vector-borne diseases are major public health concerns for billions of people in tropical and subtropical regions. Leishmaniasis is one of the most significant tropical diseases, causing about 1 million new cases and 30,000 deaths each year worldwide, and leaving hundreds of thousand others with debilitating scars. It is caused by infection by *Leishmania*, a protozoan parasite, transmitted by the bite of a female sandfly (*Phlebotomus*, *Lutzomia*)^1^. A flagellated form of the parasite, the promastigote, develops in the sandfly. Upon a blood meal of the insect, the promastigotes are transferred into the circulation of the mammalian host. Within minutes after the bite parasites are taken up by phagocytic cells, including macrophages and neutrophils, a critical step in the course of the infection^2^. The primary entry mechanism of *Leishmania* parasites into their host cells was described to be through attachment to cell surface receptors, such as the complement receptor 3 (CR3, also called CD11b) and the mannose receptor (MR, also called CD206)^3^. Short-lived neutrophils constitute an early *Leishmania* host. Then, apoptotic neutrophils are taken up by macrophages, the final resident cells of the parasites, where they initiate their intracellular development and replication, in a non-flagellated form called amastigote^4^. However, the role of neutrophils is not fully understood, and seems to depend on the parasite species and the host^2^. In any case, in these early stages, parasitic infection induces innate immune cell recruitment to the site of infection^5^.

*Leishmania* parasites are well known to take advantage of host mechanisms and to divert them to their own advantage. While internalized by phagocytes *Leishmania* hijacks host innate immune responses, inhibiting microbicidal functions and taming down inflammatory events by altering various signalling pathways^4, 6^. This favours the establishment, division and propagation of intra-macrophage dividing parasites to other naive cells.

Interestingly, the sandfly, vector of the *Leishmania* parasites, was reported to show nocturnal biting activity, from around dusk and extending into the night^7–11^. Consequently, promastigotes are more likely to be injected into the mammalian hosts at specific times of the day. This suggests that the success of the infection by *Leishmania* might be optimized according to the time of day and to the host’s endogenous daily rhythms.

Various physiological processes present ~24 h rhythms. These circadian rhythms are generated by endogenous clocks, located in most cell types. Within each cell, the circadian clock mechanism consists of clock genes organized in autoregulatory feedback loops^12^. In the main loop, the transcription factors CLOCK and BMAL1 activate expression of *Period (Per)* and *Cryptochrome (Cry)* genes, and after a time delay, the PER and CRY proteins feedback negatively on their own expression. Mutation of clock genes alters circadian rhythms^12^. For example, the deletion of *Bmal1* in mice leads to arrhythmicity. The clock controls cellular rhythms by regulating the transcription of thousands of so-called clock-controlled genes, which present rhythms in their mRNA levels^12^. Any given organ or cell type has 5 to 20% of its transcriptome showing 24 h rhythms.

As other cell types, immune system cells harbour circadian clocks^13^. This is the case for macrophages, which were shown to express numerous genes with a circadian rhythm, including genes that encode cytokines (e.g. interleukin-1) and chemokines (e.g. MIP-1α, MCP-1), and factors involved in phagocytosis and in signalling pathways responding to endotoxin and leading to cytokine gene expression^14–16^. Accordingly, various functions of macrophages were found to vary as a function of time of day, including phagocytosis, the cell’s recruitment to infected tissue, their response to microbial molecules, and the subsequent synthesis and release of cytokines^13, 15^. Neutrophil functions were also shown to be under circadian regulation. In particular, their recruitment to infected lung tissue is regulated by the local clocks and by the corticosterone rhythm^17^.

The pervasiveness of circadian rhythms in the immune system, in particular in both cell types that host *Leishmania* parasites, neutrophils and macrophages, led us to hypothesize that the infectivity of *Leishmania* presents a circadian rhythm, and that this rhythm depends on clocks within cells of the immune system. We tested these hypotheses using *in vitro* and *in vivo* models of infection, by measuring the outcome of *Leishmania* infection at different times of day, as well as upon disruption of the clock in immune cells.

## Results

### The magnitude of *L*. *major* infection is regulated by the circadian clock *in vivo*

**Figure 1:**
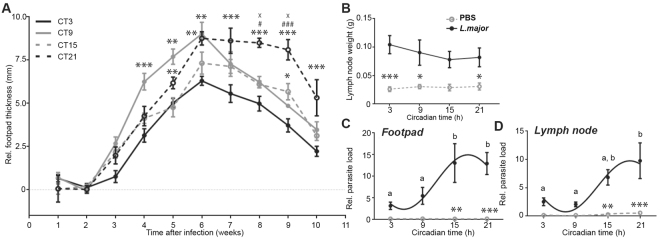
*Leishmania major* infection *in vivo* presents a circadian rhythm. (**A**) Mice were infected in the footpad during the subjective day at circadian time (CT)3 and CT9 and during the subjective night at CT15 and CT21. Footpad thickness was measured weekly; the difference between the infected and the uninfected paw is shown. Significance compared to CT3: *p < 0.05, **p < 0.01, ***p < 0.001; compared to CT9: ^#^p < 0.05, ^###^p < 0.001; compared to CT15: ^X^p < 0.05. (**B**) The weight of infected (*L*. *major*) and non-infected (PBS) draining popliteal lymph nodes was measured 10 weeks post-infection. (**C**,**D**) The parasite load was determined 10 weeks post-infection by quantifying *L*. *major* DNA by quantitative PCR in the footpad (**C**) and draining popliteal lymph node (**D**). Significant rhythms are illustrated with fitted cosine curves, otherwise data are simply connected by straight lines between data points, indicating no significant cosine fit. Significance between the two groups is indicated by stars: *p < 0.05, **p < 0.01, ***p < 0.001. Different letters indicate significant differences between time points within the *L*. *major* group. Data are presented as mean ± SEM. For details of statistics, see Supplementary Table S3.

To test whether *Leishmania* infection intensity is gated by the circadian clock *in vivo*, we injected *L*. *major* promastigotes in the footpad of C57BL/6 J mice, which caused chronic cutaneous leishmaniasis^18^, at different circadian times. We found that footpad swelling varied as a function of the time of infection (Fig. 1A). Reduced and slowest footpad swelling was measured when infection was performed in the beginning of the subjective day (circadian time [CT] 3; CT0 to CT12 is the subjective day, equivalent to the light period in the previous light:dark cycle, i.e. the resting period of the mice, whereas CT12 to CT24/0 is the subjective night, i.e. the activity period). In contrast, fastest swelling was observed when mice were infected in the end of the subjective day (CT9). Infection during the subjective night (CT15, 21) caused intermediate footpad swelling. Interestingly, infection at CT21 led to more pronounced swelling 6 weeks after infection and until the end of the experiment, while infection at CT9 and CT15 caused intermediate swelling. Nevertheless, infection at CT3 caused lowest footpad swelling throughout the entire experiment.

Lymph nodes on the side of infection weighed more than those of the non-infected side (Fig. 1B) (as previously reported^19^), but did not vary as a function of time of day. In contrast, parasite load measured using PCR on genomic DNA extracted from the footpad (Fig. 1C) and the draining popliteal lymph node (Fig. 1D) showed lowest parasite burden following infection at CT3, thus paralleling footpad swelling (Fig. 1A).

### *L*. *major* parasite attachment to macrophages is regulated by the circadian clock in these cells

**Figure 2:**
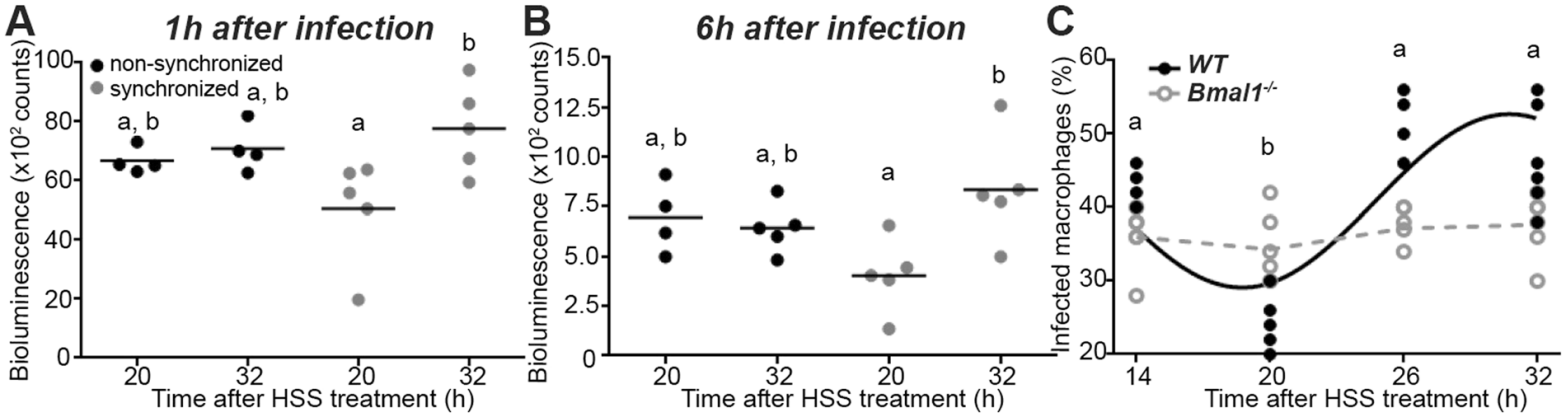
*Leishmania major* infection is regulated by the circadian clock in macrophages *in vitro*. (**A**) *L*. *major-LUC* bioluminescence 1 h after infection (i.e., promastigote attachment) of non-synchronized BMDMs or 20 h/32 h after HSS. (**B**) *L*. *major-LUC* bioluminescence 6 h after infection (i.e., promastigote internalization) of non-synchronized BMDMs or 20 h/32 h after HSS. (**C**) Promastigote attachment to BMDMs of WT or *Bmal1*-deficient mice 1 h after infection 14, 20, 26 or 32 h following a HSS. In C, significant rhythms are illustrated with fitted cosine curves, otherwise data are simply connected by straight lines between data points, indicating no significant cosine fit. Different letters indicate significant differences between time points. For details of statistics, see Supplementary Table S3.

Macrophages are the final host cells of *Leishmania* parasites^4^. Thus, we evaluated whether the observed variations in parasite burden are a consequence of changes in the infectivity of parasites due to circadian rhythms in macrophages. Indeed, it was shown that macrophages harbour their own intrinsic circadian clock, which regulates genes implicated in various macrophage functions^13, 14^. Like for other cell types, the clocks in individual macrophages can be synchronized using treatments such as a serum shock. In order to address whether the circadian clock in macrophages has an impact on their capability to get infected by *Leishmania* parasites, we compared *L*. *major* promastigote attachment to bone marrow derived macrophages (BMDMs) infected at different circadian times *in vitro*. BMDMs from PER2::Luciferase (PER2::Luc) knock-in mice showed robust circadian rhythms for at least 70 h following synchronization of the cellular clocks with a horse serum shock (HSS) (Supplementary Fig. S1A). We used luciferase-expressing *L*. *major* (*L*. *major*-Luc) promastigotes to monitor parasite attachment to and internalization in macrophages (note that PER2::Luc macrophages were not used for these experiments). Attachment was monitored by measuring bioluminescence 1 h after infection. *L*. *major*-Luc promastigotes were added to the cultured BMDMs 20 h or 32 h after HSS. Significantly fewer BMDMs showed parasite attachment when promastigotes were added to the culture 20 h after HSS, compared to 32 h (Fig. 2A). As expected, non-synchronized BMDMs (i.e. conditions where the clocks of cells in the culture were all at different phases) showed no difference in parasite attachment between the time points. Consistently, levels of bioluminescence measured 6 h after infection — reflecting internalized *L*. *major*-Luc promastigotes — also depended on time of infection in synchronized, but not in non-synchronized BMDMs (Fig. 2B). These results were confirmed by visually counting the *Leishmania* promastigotes attached to BMDMs 1 h after infection at 4 different time points after HSS (Fig. 2C, closed symbols): once again, the lowest attachment was found when the parasites were added to the culture 20 h post-HSS. This demonstrate that the attachment of *L*. *major* promastigotes to BMDMs and their internalization are regulated in a circadian manner.

To test whether the circadian clock within macrophages underlies the observed time dependence in promastigote attachment, the experiment was also done using BMDMs from mice with a knockout of the *Bmal1* gene, a critical component of the molecular circadian clock^12^. Clock disruption in BMDMs from *Bmal1* ^−/−^ mice (Fig. 2C, open symbols) abolished the circadian variation in macrophage infection. Thus, the circadian clock in macrophages is required for the circadian rhythm of attachment of *L*. *major* to these cells.

The complement receptor 3 (CD11b) and the mannose receptor (CD206), were both reported to be implicated in the attachment and internalization of *Leishmania* parasites^3^. We found both receptors to be rhythmically expressed at the surface and inside of non-infected BMDMs after HSS (Supplementary Fig. S1B–E) and of non-infected and infected BMDMs (harvested 1 h after infection) from control mice (Supplementary Fig. S2). Of note, the infection affected the expression levels in a time-dependent manner, shifting the peak of expression (Supplementary Fig. S2A,B). Importantly, the rhythmic expression of CD206 and CD11b was absent in BMDMs from *Bmal1* ^−/−^ mice (Supplementary Fig. S2C,D), indicating that these receptors are regulated by the macrophage circadian clock. Unexpectedly though, the acrophase (time of peak) of promastigote attachment did not align with the acrophase of CD206 and CD11b expression in BMDMs (compare phases of rhythms in Fig. 2C and Supplementary Fig. S1B–E), suggesting that the rhythms of these receptors are not responsible for the observed circadian rhythm in attachment.

### Circadian immune cell recruitment to the infection site after *L*. *major* infection

**Figure 3:**
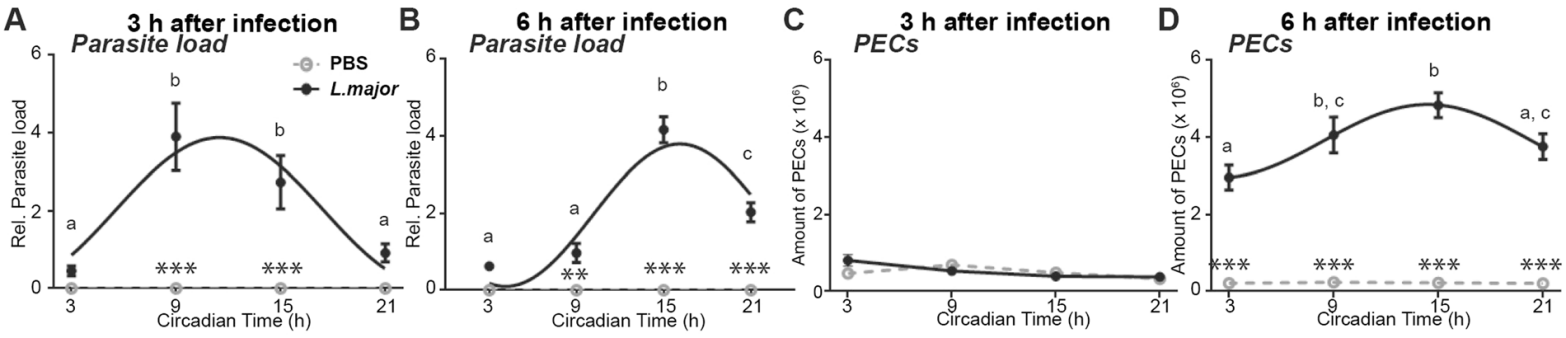
*Leishmania major* parasite load and peritoneal exudate cell (PEC) numbers present circadian rhythms *in vivo*. The parasite load (**A**,**B**) and PEC numbers (**C**,**D**) in *L*. *major*-infected (and PBS-injected control) mice during the circadian day were determined 3 h (**A**,**C**) or 6 h (**B**,**D**) post-infection by quantifying the *L*. *major* DNA by quantitative PCR on PEC DNA and by counting PECs, respectively. Significant rhythms are illustrated with fitted cosine curves, otherwise data are simply connected by straight lines between data points, indicating no significant cosine fit. Significance between the two groups are indicated by stars: **p < 0.01; ***p < 0.001. Different letters indicate significant differences between time points within the *L*. *major* group. Data are presented as mean ± SEM. For details of statistics, see Supplementary Table S3.

As indicated above, the circadian expression of receptors is unlikely to account for the rhythmic parasitic infection of macrophages *in vitro*. Thus, to evaluate whether circadian rhythms in the presence of immune cells may be implicated in the circadian magnitude of infection *in vivo*, we compared immune cell frequencies 3 h and 6 h after injection of *L*. *major* promastigotes in the peritoneal cavity of C57BL/6 J mice at different circadian times. Although injection in the skin would be a closer mimic of the sandfly bite inoculation, peritoneal injection is a widely used model of *Leishmania* infection in mice that is particularly suitable to study the acute recruitment of innate immune cells to the site of infection. Consistent with our previous results obtained after footpad infection (Fig. 1), parasite load of peritoneal exudate cells (PECs) showed circadian rhythmicity when measured 3 h and 6 h after infection with peak levels when infection was at CT9 or CT15 (Fig. 3A,B). Moreover, lowest parasite load was detected when infection was done in the morning (CT3). Although recruitment of immune cells was undetectable 3 h after infection (Fig. 3C), a circadian rhythm of recruitment was observed after 6 h (Fig. 3D). Consistent with the peak levels of parasite load, strongest immune cell recruitment was observed after infection at CT15 (Fig. 3D).

**Figure 4:**
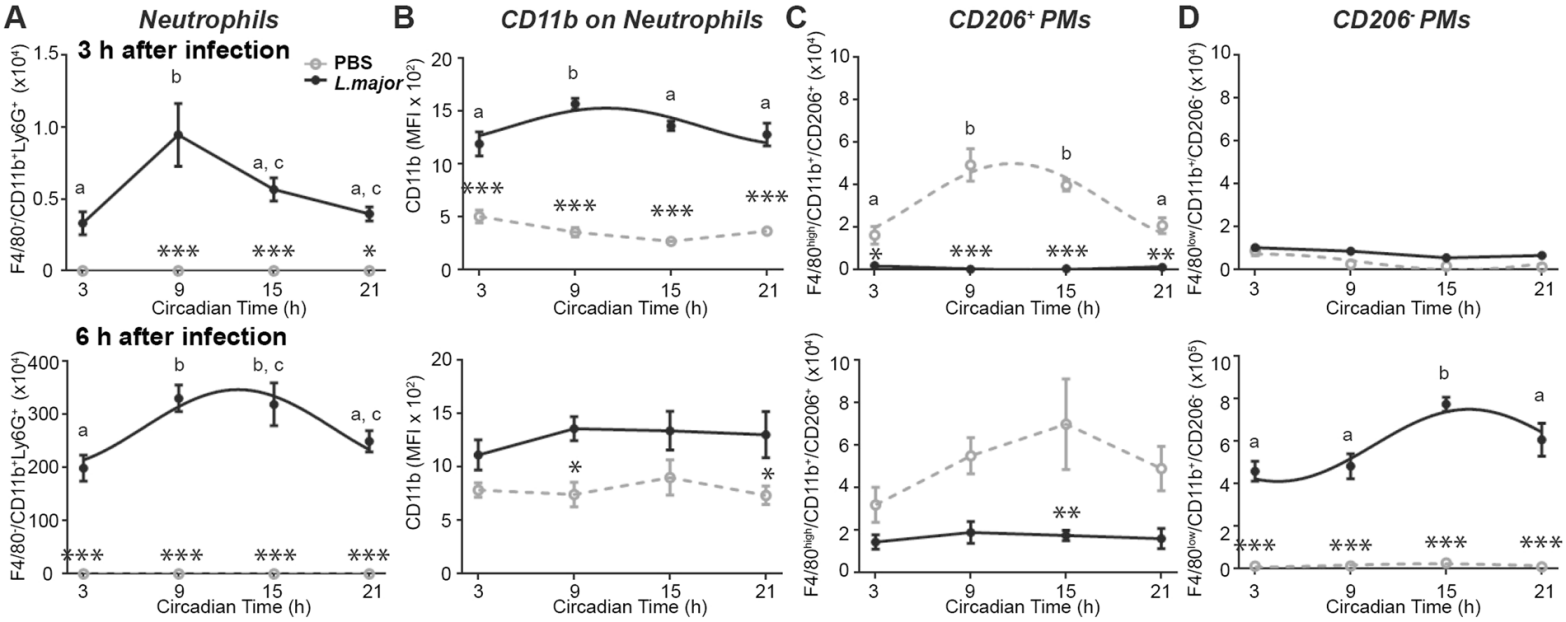
Circadian immune cell recruitment after *Leishmania major* infection *in vivo*. (**A**,**B**) Frequencies of recruited F4/80^−^CD11b^+^Ly6G^+^ neutrophils (**A**) and mean fluorescence intensity (MFI) of CD11b on neutrophils (**B**) were determined in peritoneal exudate cells (PECs) 3 h (**top**) or 6 h (**bottom**) after injection with PBS or *L*. *major* in the peritoneal cavity of mice over the circadian day. (**C**,**D**) Frequencies of F4/80^high^CD11b^+^CD206^+^ peritoneal macrophages (PMs) (**C**) and F4/80^low^CD11b^+^CD206^−^ PMs (**D**) either 3 h (**top**) or 6 h (**bottom**) after injection of mice with PBS or *L*. *major* over the circadian day. Significant rhythms are illustrated with fitted cosine curves, otherwise data are simply connected by straight lines between data points, indicating no significant cosine fit. Significance between the two groups are indicated by stars: *p < 0.05; **p < 0.01; ***p < 0.001. Different letters indicate significant differences between time points within each group. Data are presented as mean ± SEM. For details of statistics, see Supplementary Table S3.

Characterization of cell frequencies among PECs in non-infected mice revealed that the recruitment of neutrophils varied according to the time of infection with minimal recruitment during the early subjective day (CT3) and peak levels at CT9 or between CT9 and CT15 when measured 3 h or 6 h after infection, respectively (Fig. 4A). CD11b was shown to facilitate the uptake of *Leishmania* parasites into neutrophils^20^. CD11b expression was increased in these cells 3 h after infection and followed a circadian pattern similar to the parasite load (compare Figs 3A with 4B). However, this rhythm was absent 6 h after infection.

Interestingly, a circadian rhythm in the frequency of pro-inflammatory CD206^+^ peritoneal macrophages (PMs) was found in control mice, but was suppressed in infected mice (Fig. 4C). In contrast, the frequency of anti-inflammatory CD206^−^ PMs was low in control mice and 3 h after infection, but strongly induced 6 h after infection with a circadian profile (Fig. 4D) similar to corresponding parasite load (Fig. 3A,B). In contrast to results obtained on BMDMs *in vitro*, we did not find rhythmic receptor expression on PMs *in vivo* (Supplementary Fig. S3). Of note, CD11b receptor on CD206^−^ PMs was upregulated 3 h but not 6 h after infection (Supplementary Fig. S3E,F).

Although CD4^+^ T cell frequency exhibited a circadian rhythm in control mice, this rhythm was lost in infected mice (Supplementary Fig. S4A,B). Nevertheless, more CD4^+^ T cells were found 6 h after infection. No rhythms of CD8^+^ T cell frequency were detected in control or infected mice. Similar to results obtained for CD4^+^ T cells, the frequency of CD8^+^ T cells was induced 6 h after infection. However, CD8^+^ T cell frequency was significantly higher in mice infected at CT9 than in those infected at CT3 (Supplementary Fig. S4C,D).

Taken together these results indicate a circadian regulation of immune cell recruitment to the site of infection, in particular neutrophils and to a lesser degree CD206^−^ PMs, the primary and final *Leishmania* host cells^4, 21^.

### The circadian clock in immune cells regulates the magnitude of *L*. *major* infection

**Figure 5:**
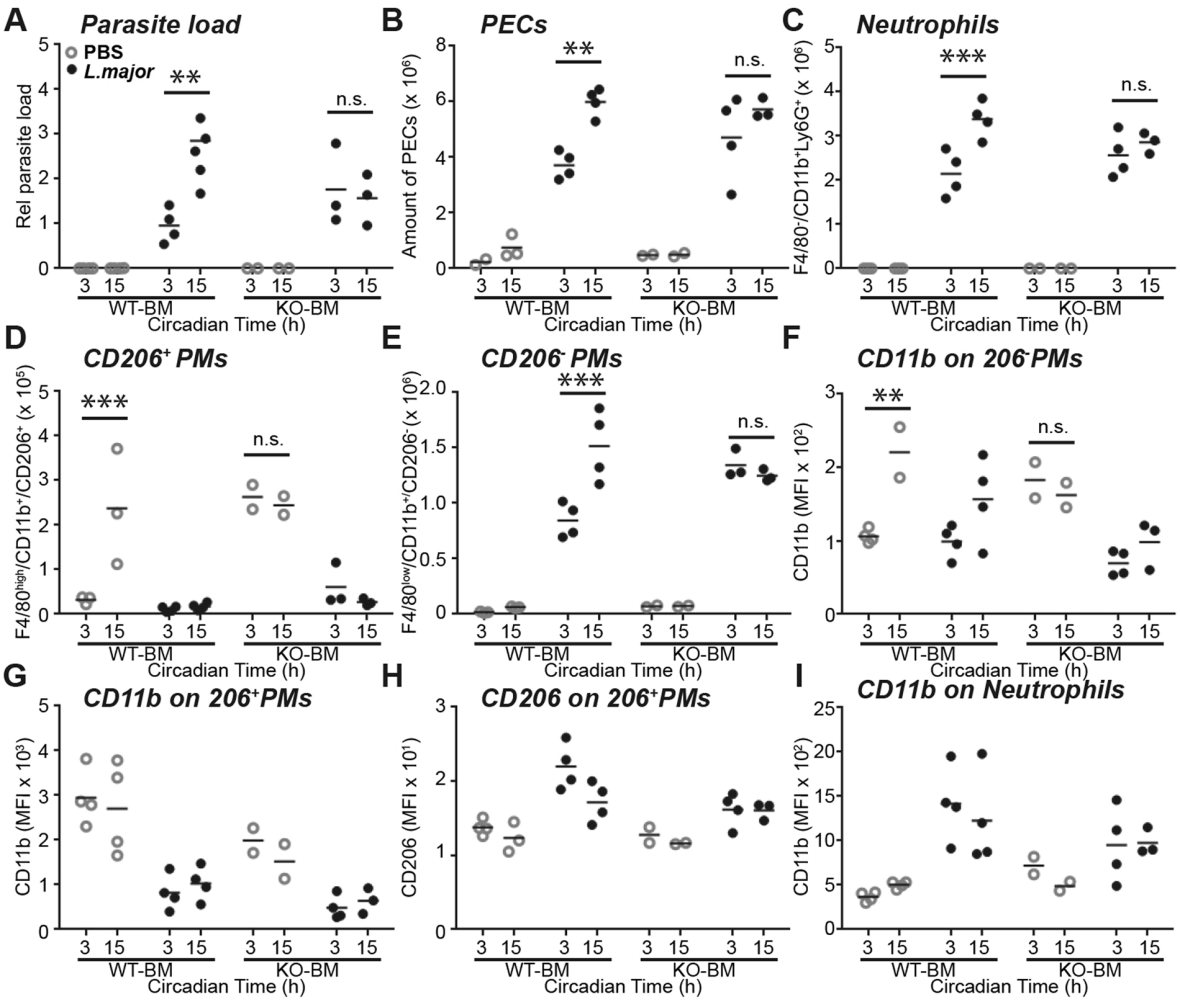
Parasite load and immune cell recruitment after *Leishmania major* infection is controlled by the circadian clock in phagocytic immune cells *in vivo*. (**A**,**B**) Parasite load (**A**) and total mouse peritoneal exudate cells (PECs) frequencies (**B**) were determined by quantitative PCR and flow cytometry 6 h after *L*. *major* infection or PBS injection performed at CT3 or CT15 in chimeric mice consisting of irradiated B6.SJL hosts that have received bone marrow cells from either *Bmal1* ^+/+^ OT-1 *Rag2* ^−/−^ (WT-BM) or *Bmal1* ^−/−^ OT-1 *Rag2* ^−/−^ donors (KO-BM). (**C–E**) Frequencies of specific cell types in the same mice: F4/80^−^CD11b^+^Ly6G^+^ neutrophils (**C**), F4/80^high^CD11b^+^CD206^+^ peritoneal macrophages (PMs) (**D**) and F4/80^low^CD11b^+^CD206^−^ PMs (**E**). (**F–I**) Mean fluorescence intensity (MFI) of CD11b on CD206^−^ PMs (**F**), 206^+^ PMs (**G**), CD206 on 206^+^ PMs (**H**) and CD11b on neutrophils (**I**) in PECs of the same mice. Significant differences between the time points are indicated by stars: **p < 0.01; ***p < 0.001. For details of statistics, see Supplementary Table S3.

We then set out to evaluate whether the circadian clock in immune cells recruited at the site of infection plays a role in regulating the magnitude of *L*. *major* infection and their own recruitment. We repeated the above experiments in mice lacking the essential clock gene *Bmal1* in neutrophils and macrophages. To this end, bone marrow from either *Bmal1* ^+/+^ (WT-BM) or *Bmal1* ^−/−^ (KO-BM) *Rag2*-deficient donor mice, which intrinsically lack mature T and B lymphocytes^22^, was used to reconstitute hematopoietic cells of irradiated B6.SJL congenic recipient mice. The frequency of CD45.2 (donor) cells was close to 100% for neutrophils and monocytes (CD45.2/[CD45.2 + CD45.1] > 0.98), demonstrating the efficiency of the bone marrow depletion and graft. As expected, in WT-BM mice 6 h after *L*. *major* infection at CT15 led to a higher parasite load than when it was done at CT3 (Fig. 5A), which confirmed previous results obtained using C57BL/6 J mice (Fig. 3B). The recruitment of PECs was also higher after infection at CT15 than CT3 in WT-BM mice (Fig. 5B). The time-dependent differences in parasite load and immune cell infiltration were absent when infection was performed in KO-BM mice (Fig. 5A,B), although the efficiency of hematopoietic reconstitution was similar between genotype. Accordingly, the difference observed in neutrophil infiltration was abolished in KO-BM mice (Fig. 5C). Of note, while WT-BM mice showed the time-dependent variation of CD206^+^ PMs in non-infected mice, this variation was missing in KO-BM mice (Fig. 5D). Also, similar to neutrophils, circadian clock disruption (in KO-BM mice) abolished the time-difference in the frequency of CD206^-^ PMs (Fig. 5E) after infection and their CD11b receptor expression (Fig. 5F), whereas CD11b and CD206 expression in CD206^+^ PMs (Fig. 5G,H) and CD11b expression in neutrophils (Fig. 5I) were not affected by the loss of *Bmal1*.

In summary, clock disruption in hematopoietic cells was sufficient to abolish the circadian effect on immune cell recruitment after *L*. *major* infection, showing that the circadian clock in these cells plays a major role in gating the efficiency of parasite uptake.

### The circadian clock in immune cells controls the rhythm of chemokine and cytokine expression following *L*. *major* infection

**Figure 6:**
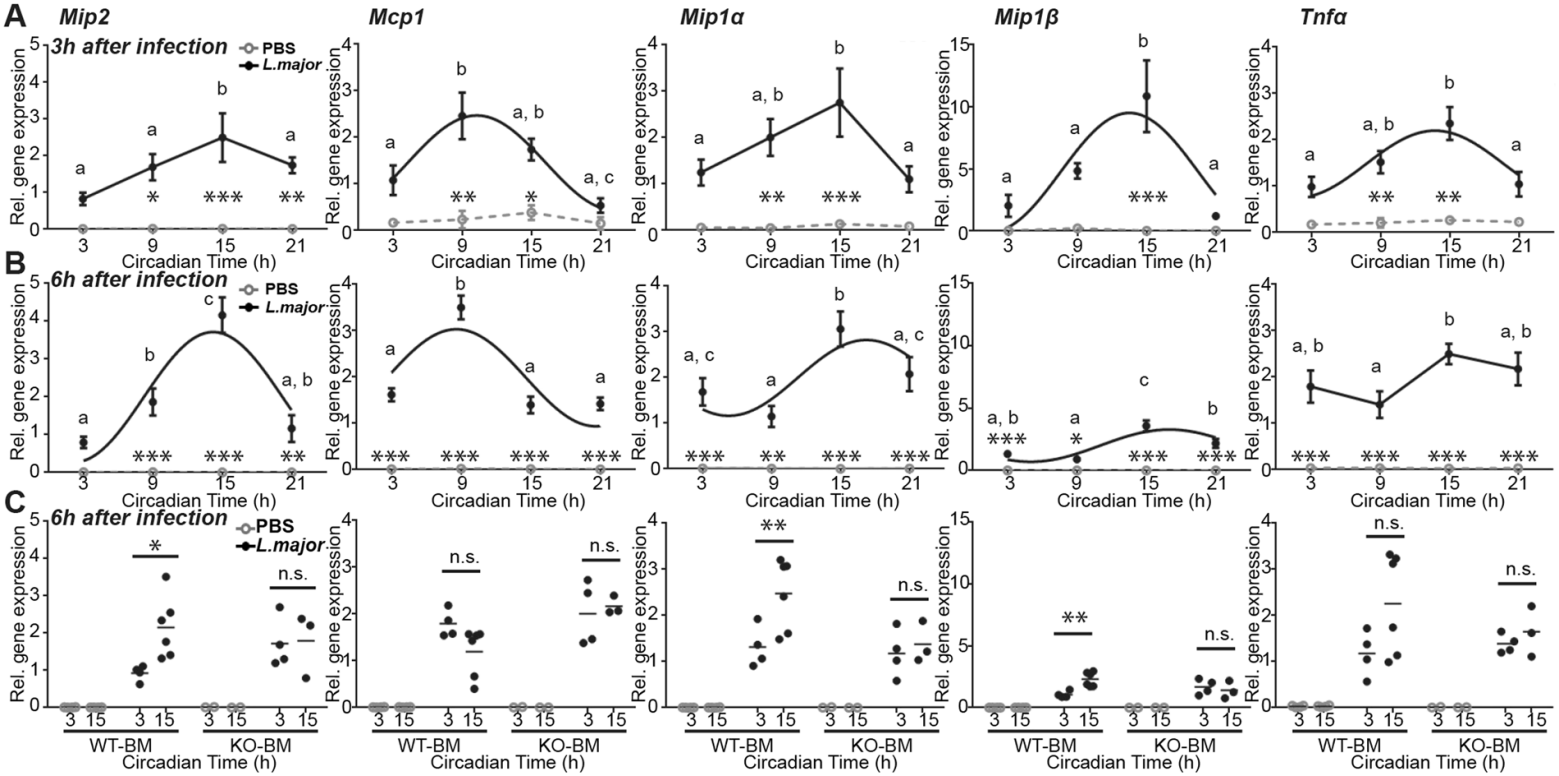
Circadian expression of chemoattractants after *L*. *major* infection *in vivo*. (**A**,**B**) *Mip2*, *Mcp1*, *Mip1α*, *Mip1β* and *Tnfα* expression in peritoneal exudate cells (PECs) of *L*. *major-*infected or PBS-injected mice 3 h (**A**) or 6 h (**B**) (n = 5) after injection. Significant rhythms are illustrated with fitted cosine curves, otherwise data are simply connected by straight lines between data points, indicating no significant cosine fit. (**C**) Cytokine/chemokine expression 6 h after *L*. *major* or PBS injection in chimeric mice consisting of irradiated B6.SJL hosts that have received bone marrow cells from either *Bmal1* ^+/+^ OT-1 *Rag2* ^−/−^ (WT-BM) or *Bmal1* ^−/−^ OT-1 *Rag2* ^−/−^ donors (KO-BM). In A and B, data are presented as mean ± SEM; Significant rhythms are illustrated with fitted cosine curves, otherwise data are simply connected by straight lines between data points, indicating no significant cosine fit; significance between the two groups are indicated by stars: *p < 0.05; **p < 0.01; ***p < 0.001. Significant differences between are indicated by stars: *p < 0.05; **p < 0.01, ***p < 0.001; different letters indicate significant differences between time points within the *L*. *major* group. In (**C**), significant differences between the time points are indicated by stars: *p < 0.05; **p < 0.01. For details of statistics, see Supplementary Table S3.

Circadian recruitment of neutrophils and PMs to the infection site may be caused by the time-dependent secretion of chemoattractants. To address this, we studied the expression of *Mip2*, *Mcp1*, *Mip1α*, *Mip1β* and *Tnfα* (Fig. 6), all known to recruit neutrophils and/or macrophages^23–28^ (expression relative to the housekeeping gene *Ef1a*). Three hours (Fig. 6A) and 6 h (Fig. 6B) after infection of C57BL/6 J mice with *L*. *major*, we found rhythmic mRNA expression for all 5 genes in PECs. Moreover, the peak expression of these chemokines matched the time of infection causing the highest parasite load and neutrophil and PM cell frequencies.

We then compared chemokine and cytokine expression profiles in mice with a *Bmal1* KO in immune cells (KO-BM) and control mice with WT bone marrow graft (WT-BM). We found again the same time-dependent variations as for C57BL/6 J mice (Fig. 6C): as in the 4 CT experiment (Fig. 6B), *Mip2*, *Mip1α* and *Mip1β* showed higher expression upon infection at CT15 than at CT3, *Mcp1* had similar level of expression for both time points, and *Tnfα* had a slightly (but not significantly) higher response to infection at CT15. These time-dependent differences were abolished when infection was performed in KO-BM mice (Fig. 6C), indicating that the clocks in neutrophils, PMs and monocytes regulate their own circadian chemokine/cytokine expression and thus, control circadian immune cell infiltration and parasite load following *L*. *major* infection.

## Discussion

In this report, we showed a circadian rhythm of the magnitude of *Leishmania major* infection in mice. Indeed parasite infectivity varied over 24 h in macrophages *in vitro* and in two different *in vivo* models, footpad and peritoneal cavity infection. This was paralleled by a daytime-dependent neutrophil and anti-inflammatory macrophage infiltration to the infection site, and rhythmic expression of chemokines attracting neutrophils and PMs. Importantly, ablating the clock in BMDMs *in vitro* and in immune cells *in vivo* abolished all rhythms. To our knowledge this is the very first report demonstrating that the circadian clock controls the infection by protozoan parasites in mammals.

The natural vector of *Leishmania* parasites – the sandfly – exhibits circadian behaviour^7, 9, 10^. *Leishmania* infection causes a strong immune response in the host and recent work including our own work indicates that the immune system is under circadian clock control^5, 13^. Therefore, we speculated that the circadian biting behaviour may have evolved to target the time of highest host sensitivity for infection. Thus, we aimed to test the hypothesis that the infection susceptibility is influenced by the host’s circadian clock. Indeed, we observed daytime-dependent infection efficiency, indicated by circadian variation in the magnitude of parasite load in cultured BMDMs. This rhythm was absent when BMDMs with a genetic clock disruption were used, showing a gating of the susceptibility of macrophages by their own circadian clock. Moreover, a circadian variation of parasite load was observed in two different *in vivo* models: infection of promastigotes in the footpad and in the peritoneal cavity of mice. Interestingly, peak levels occurred during the day-night transition, matching the documented circadian behaviour of the sandfly^7, 9, 10^. A time-dependent footpad swelling was previously observed for infection of hamsters with *L*. *amazonensis* under light:dark conditions^29^. In contrast, our study was performed in constant conditions, allowing us to conclude an endogenous nature for the rhythm. This is supported by the observation that the rhythms of infection and immune response were abolished when parasites were injected in mice lacking a functional clock in immune cells. Thus, we demonstrate that the magnitude of parasitic infection is regulated by the intrinsic, cell-autonomous circadian clocks, specifically in cells known to function as parasite hosts.

A possible cause for pronounced susceptibility to *L*. *major* infection around the day-night transition may be an increased uptake of parasites into host cells at this time of day. The main receptors implicated in the attachment and uptake of *Leishmania* are the mannose receptor (CD206) and the complement receptor 3 (CD11b)^6^. Whereas CD206 is specific for macrophages, CD11b is additionally expressed on neutrophils. We reported circadian rhythms of CD206 and CD11b on BMDMs *in vitro*, but in antiphase with the attachment rhythm, and no rhythm of CD206 was found for PMs *in vivo*. Consequently, the circadian regulation of these receptors on macrophages probably does not account for the observed circadian variation of infection. Previous reports on *L*. *major* infection in CD11b- and CD206-deficient mice indicated a minor role for these receptors in parasite susceptibility^30, 31^. Of note though, the levels of CD11b in CD206^-^ PMs *in vivo* were significantly lower at CT3 than at CT15, which parallels the magnitude of the infection. However, infection did not affect CD11b levels in these PMs. Interestingly, we did find rhythmic CD11b receptor expression on neutrophils early after infection, with peak levels occurring at the time of highest parasite infectivity. Receptor-mediated phagocytosis of parasites into neutrophils has been described^32^. Consequently, this receptor may be implicated in the rhythmic uptake of parasites and thus, the rhythmic parasite load early after infection. Of note, additional receptors have been found^6^, which may participate in the rhythmic uptake of promastigotes. Moreover, we cannot exclude a role of circadian clocks in *Leishmania* parasites themselves, as a recent report described circadian rhythms of gene expression intrinsic to the single-cell parasite *Trypanosoma brucei* ^33^.

Higher amounts of immune cells at the time of infection could be an alternative cause for a more pronounced parasitic infection. Indeed, in uninfected mice we observed more CD206^+^ pro-inflammatory macrophages at the day-night transition (CT9–15). However, *L*. *major* infection strongly reduced the amount of these macrophages or suppressed the CD206 receptor expression at their surface. Consequently, rhythms in the abundance of pro-inflammatory macrophages could cause the rhythm in chemokines/cytokines early after infection, before they get suppressed (or CD206 is downregulated). We also observed a high presence of immune cells at the day-night transition after infection. In particular, we measured a strong recruitment of neutrophils and to a lower extent of CD206^-^ anti-inflammatory PMs to the infection site. The magnitude of the cell recruitment was dependent on the time of infection. Consistently, enhanced recruitment of neutrophils and macrophages was previously documented early after infection with *L*. *major* in air pouches^26^. Neutrophils are typically the first leukocytes to be recruited to an inflammatory site, as previously documented after *L*. *major* skin, footpad or air pouch infection^5, 18, 19, 21, 34, 35^, and they represent an important early target for *Leishmania* parasites^4, 32^. Here we demonstrate a rhythmic recruitment of neutrophils to the infection site with peak levels at the day-night transition. Thus, rhythmic neutrophil recruitment is likely the underlying cause of the observed rhythmic parasite load, because neutrophils can serve as host cells in early infection stages^36^.

*Leishmania* parasitic infection induces the expression of chemotactic factors that selectively attract immune cells, specifically neutrophils and macrophages (reviewed in ref. 36) and thus may critically control the parasite load. Indeed, we measured increased expression of pro-inflammatory cytokines and chemokines such as TNF-α, MIP-1α/CCL3, MIP-1β/CCL4, MCP-1/CCL2, and MIP-2/CXCL2 in PECs rapidly after *L*. *major* infection. This is in accordance with previous reports^26, 37^. For example MIP-2 secreted by infected macrophages is an important chemokine for the recruitment of neutrophils^23^ and critical for intra-macrophage killing of *L*. *major* ^38^. TNF-α, MIP-1α, MIP-1β and MCP-1 are chemoattractants produced by neutrophils^25^. MCP-1, MIP-1α and MIP-1β mediate recruitment of macrophages, monocytes, basophils, eosinophils, NK cells and lymphocytes^24, 26–28^, important in cellular responses to *Leishmania* ^26^. MCP-1 and MIP-1α mediate macrophage activation for subsequent parasite clearance, and thus may play a role in the containment of *Leishmania* infection^39^. The circadian expression of MIP-1α and MIP-1β may account for the circadian response of CD8^+^ T cells observed 6 h after infection, because the induction of MIP-1α/β was suggested to play an important role in lymphocyte accumulation during the adaptive immune response to *L*. *major* ^26^.

Importantly, the inducibility of all cytokines/chemokines examined was dependent on the time of infection. Moreover, the peak levels of the rhythmic chemoattractant expression occurred at the same circadian time as the highest immune cell frequencies and susceptibility to the parasite. Previous reports showed a circadian rhythm of chemokines, such as MCP-1^15, 16, 40^ and CXCL5^17^, and corresponding rhythms of cell trafficking to different tissues^16, 17, 40^. Our work is the first to show that such a regulation is relevant in the context of parasitic infection. Importantly, the observed circadian rhythms in parasite load, immune cell recruitment and cytokine/chemokine expression were all abolished in mice lacking the circadian clock in immune cells including neutrophils and macrophages, indicating that the intrinsic circadian clock in phagocytic cells is the source of the observed rhythmic susceptibility to the parasite.

**Figure 7:**
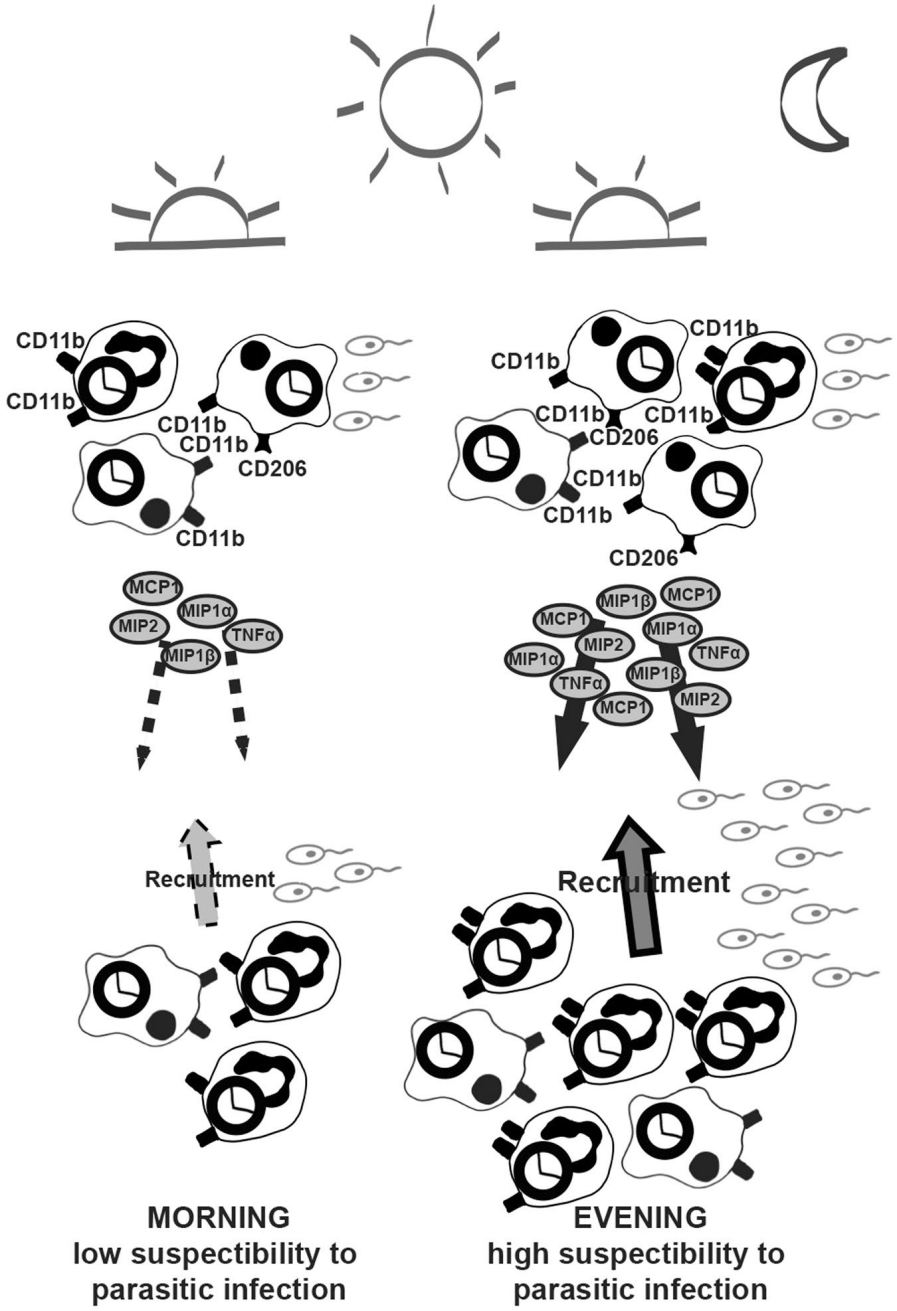
Circadian rhythm of *L*. *major* parasitic infection in mammals. The circadian clock controls the number of phagocytic host cells exposed to the parasites and their surface receptor expression. In the evening, higher amounts of CD206^+^ macrophages circulate through the bloodstream and there are more CD11b receptors present on neutrophils. Additionally, the secretion of chemokines/cytokines is controlled by the circadian clock. *L*. *major* infection during the evening induces higher level of chemoattractants which in turn elevates the recruitment of neutrophils and CD206^-^ macrophages. Consequently, more host immune cells can become invaded by parasites, leading to a higher magnitude of infection in the evening.

Collectively our findings show for the first time a circadian rhythm of protozoan parasitic infection in mammals (Fig. 7). The circadian clock controls the number of phagocytic cells circulating through the bloodstream and the receptor expression on their surface which are relevant for the attachment and internalization of parasites. When the parasitic infection occurs during the early night, higher amounts of host cells (CD206^+^ macrophages) and the receptors (CD11b on neutrophils) are present. Moreover, the chemokine/cytokine secretion and the subsequent recruitment of neutrophils and CD206^-^ macrophages is regulated by the circadian clock within immune cells. Upon neutrophil and macrophage infection during the night, their chemokine/cytokine secretion is elevated. Accordingly, parasitic infection during the night induces a stronger recruitment of neutrophils and CD206^-^ macrophages. Considering that neutrophils and macrophages are cellular hosts of *Leishmania* parasites, an increased recruitment during the night can consequently lead to an enhanced host’s susceptibility to the parasitic infection, hence the increased parasite load we observed.

In summary, the circadian clock in immune cells regulates their susceptibility to become infected and regulates their recruitment following *L*. *major* infection by controlling the expression of chemoattractants and thus rhythmically gates *Leishmania* infection efficiency. Thus, our work reveals that the host’s circadian clocks have a strong impact on *Leishmania* infection. A better understanding of the timed regulation of host-parasite interactions will allow developing better prophylactic and therapeutic strategies to fight-off vector-borne diseases.

## Methods

### Cell culture

BMDMs were derived from mononuclear phagocyte progenitor cells flushed from femurs and tibias of C57BL/6 J, PER2::LUC (B6.129S6-Per2^tm1Jt^/J, Jackson Laboratories) and *Bmal1* knockout mice (from Dr. K.F. Storch) and cultured using standard conditions. *L*. *major* LV39 (MRHO/Sv/59/P) and *L*. *major-Luc* were grown in SDM-79 medium and cultured using standard conditions. See Supplementary Methods for details.

### *L. major* infection *in vitro*

BMDMs were infected with stationary-phase promastigotes (as opposed to metacyclic promastigotes) in a 20:1 *Leishmania*-BMDM ratio, a dose similar to that of other reports^35, 41, 42^, and incubated at 37 °C with CO_2_. Unattached and non-internalized parasites were removed 1 h after infection by washing the plates with PBS. A total of 100 BMDMs were counted in a hemocytometer to evaluate attachment. In other experiments, PBS washes were done 1 h and 6 h after infection to detect attachment and internalization of *L*. *major-Luc* promastigotes by bioluminescence recording in sealed dishes in a LumiCycle luminometer (Actimetrics Inc.). These experiments have been performed three times, with similar outcomes.

### *L*. *major* injections and footpad growth monitoring

Animal use was in accordance with the guidelines of the Canadian Council of Animal Care and was approved by the Douglas Institute Facility Animal Care Committee. C57BL/6 J mice, B6.SJL mice, *Bmal1* ^−/−^ mice and *Bmal1* ^−/−^ OT-1 *Rag2* ^−/−^ mice (Taconic) and WT littermates were housed (≤5/cage) under 12 h light (100 lux):12 h dark (0 lux) (LD) conditions with food and water available *ad libitum*. Male mice (2–3 months old) were used in most experiments, except to evaluate footpad growth, where 3–4 months old female mice were used, and for bone marrow grafts (see below).

Five million stationary-phase parasites in 50 µL were injected in the left and PBS in the right hind footpad of C57BL/6 J mice. This dose is high compared to what is deposited by the sand fly during a blood meal, but it is similar to that used in other reports^43–45^. Footpad swelling was measured daily with a calliper and calculated as the difference of the thickness between both footpads. This experiment has been performed twice, with similar outcomes. In other experiments, C57BL/6 J mice or B6.SJL mice were inoculated in the peritoneal cavity with either 10^7^ (PEC collection 3 h after infection) or 10^8^ (PEC collection 6 h after infection) *L*. *major* promastigotes. This was performed at 4 time points in wild-type (C57BL/6 J) mice and at 2 time points in bone marrow-grafted mice (see below), with similar outcomes at comparable time points.

### Tissue collection

Mice were sacrificed by cervical dislocation at the indicated times after inoculation in the second day of darkness. Footpads and lymph nodes were harvested after 10 weeks and spleen and PECs 3 h or 6 h after the *L*. *major* infection and kept on ice (flow cytometry) or at −80 °C (for quantitative PCR). Splenocytes were homogenized in RPMI using a cell strainer (Thermo Fisher). Cells were then incubated for 5 min at RT with 0.83% NH_4_Cl to lyse the red blood cells and washed with RMPI. Cells were fixed in 4% paraformaldehyde for 20 min at room temperature, washed in PBS, and stored at 4 °C until further staining.

### Bone marrow transplantation

B6.SJL host mice (12–30 weeks old) with a CD45.1 allele received antibiotics for 3 days before the irradiation procedure. Host mice received 950 rad whole-body lethal x-ray irradiation (RAD SOURCE Technologies RS2000) to achieve total ablation of host hematopoietic tissues. Immediately after the irradiation host mice received 10^7^ bone marrow cells from *Bmal1* ^+/+^ OT-1 *Rag2* ^−/−^ or *Bmal1* ^−/−^ OT-1 *Rag2* ^−/−^ mice (8–10 weeks old) with an isogeneic CD45.2 C57BL/6 background. *L*. *major* infection was done after 30 days. See Supplementary Methods for details.

Quantitative PCR, immune cell frequency and receptor expression were performed using standard protocols (see Supplementary Methods and Supplementary Tables S1 and S2).

### Statistical analysis

Statistical analyses were done with GraphPad Prism (GraphPad Software). Circadian variation was tested by fitting a cosine wave equation y = B + (A * cos (2 * *π* * ((x − Ps)/24))) on gene expression data, where B is the baseline, A is the amplitude, Ps is the phase shift, with a fixed 24-h period; significance was determined by F-test. For differences over time between 2 or more groups two-way ANOVA was used, followed by Bonferroni’s multiple comparison posthoc test. A statistically significant difference was assumed when p < 0.05.

### Data availability

The datasets generated during the current study are available from the corresponding author on reasonable request.

## Electronic supplementary material

Supplementary Material
