# Supplementary material for: The circadian clock in immune cells controls the magnitude of Leishmania parasite infection
Source: Sci Rep. 2017 Sep 7;7:10892. doi: 10.1038/s41598-017-11297-8 (PMC5589941; doi:10.1038/s41598-017-11297-8)
Supplement: Supplementary file 1 — Supplementary Material [file 41598_2017_11297_MOESM1_ESM.pdf]

The circadian clock in immune cells controls the magnitude of *Leishmania* parasite infection

Silke Kiessling, Geneviève Dubeau-Laramée, Hyejee Ohm, Nathalie Labrecque, Martin Olivier and Nicolas Cermakian

Supplementary Material

Supplementary Methods

Cell culture. BMDMs were derived from mononuclear phagocyte progenitor cells flushed from femurs and tibias of C57BL/6J, PER2::LUC (B6.129S6-Per2^{tm1Jt}/J, Jackson Laboratories) and *Bmal1* knockout mice (from Dr. K.F. Storch) and cultured in the presence of M-CSF and of L929 cell conditioned medium (LCCM), obtained by culturing L929 cells in RPMI/10% FBS at 37°C. BMDMs were cultured in RPMI with 10% FBS, 50 U/mL penicillin, 50 µg/mL streptomycin and 30% LCCM at 37°C. The serum shock procedure was performed as described, by treating the BMDMs with 50% horse serum in RPMI for 2 h, and then replacing it with serum-free medium (1). Cells were stained for F4/80 and analysed by flow cytometry to ensure that at least 99% cells were macrophages (F4/80$^+$).

L. major and *L. major-Luc* were grown in SDM-79 medium with 10% heat-inactivated FBS and hemin (5 mg/ml) in a dry incubator at 25°C. The parasites were split in a 1:20 ratio every 3 days when parasites were in their log growth phase. All culture reagents were from Life Technologies.

Bone marrow transplantation. B6.SJL host mice (12-30 weeks old) with a CD45.1 allele received antibiotics for 3 days before the irradiation procedure. Host mice received 950 rad whole-body lethal x-ray irradiation (RAD SOURCE Technologies RS2000) to achieve total ablation of host hematopoietic tissues. Donor bone marrow cells were obtained from the femoral and tibial bones of *Bmal1*$^{+/+}$ OT-1 *Rag2*$^{-/-}$ or *Bmal1*$^{-/-}$ OT-1 *Rag2*$^{-/-}$ mice (8-10 weeks old) with an isogeneic CD45.2 C57BL/6 background. Immediately after the irradiation host mice received 10^7 bone marrow cells in PBS through injection in the tail vein. *L. major* infection was done after 30

days. Splenocytes were assessed by flow cytometry for the percentage of recipient CD45.1 and donor CD45.2 leukocytes using standard techniques described below. Engraftment was calculated by the ratio [CD45.2$^+$ cells / (CD45.2$^+$ cells + CD45.1$^+$ cells)] and was on average 0.98 ± 0.004 (neutrophils) 0.99 ± 0.001 (macrophages and monocytes), 0.81 ± 0.01 (T cells) and 0.89 ± 0.02 (B cells). There are no T and B cells in the graft because it originates from *Rag2*$^{-/-}$ mice, so the T/B cell precursors that may have remained in the host mice have partly replenished T and B cells in the mice (with CD45.1$^+$). The graft being from OT-1 mice, they have CD8+ T cells, explaining the presence of CD45.2$^+$ T cells.

Immune cell frequency and receptor expression. One million PECs were washed with PBS and blocked with Fc Block (#101320, Biolegend) on ice for 10 min, washed and incubated on ice for 20 min with: biotin anti-CD11b, APC anti-F4/80 and FITC anti-Ly6G to measure frequency of neutrophils; APC anti-F4/80, biotin anti-CD11b and anti-CD206 for PMs; AF647 anti-CD3, PE anti-CD4, PerCP anti-CD8, FITC anti-CD44 for T cells; and AF647 anti-CD19 for B cells. For CD11b staining, cells were washed with PBS and incubated with PerCP-conjugated streptavidin for 20 min on ice. Antibodies are listed in Supplementary Table S1. Finally cells were washed with PBS or PBS/1% BSA and stored at 4°C.

Receptor expression on BMDM was measured using the same protocol to detect cell surface expression and cells were fixed with 4% PFA for 20 min and permeabilised with 90% methanol prior to staining to detect intracellular receptor expression.

The samples were analysed by flow cytometry using a FACSCalibur (BD Biosciences) and FlowJo software (FlowJo, LLC). Examples of gating strategy for each staining are shown in Supplementary Figure S5.

Quantitative PCR. Total RNA from PECs was isolated using Trizol (Life Technologies). RNA concentration and purity were determined with an ND-1000 spectrophotometer (Thermo Fisher). Complementary DNA was synthesized using the High Capacity cDNA Reverse Transcription Kit (Applied Biosystems). Quantitative PCR was performed in a 7500 Real Time PCR System (Life Technologies) using GoTaq qPCR Mastermix (Promega). Protocols from manufacturers were used. *Elongation factor 1-alpha (Ef1α)* was used as control gene and relative quantification of expression levels were performed as described (2). *L. major* parasite load was evaluated by quantitative PCR as described (3). Footpads, lymph nodes or PECs were digested at 55°C in SNEP buffer containing Proteinase K (20 ng/mL). Genomic DNA was extracted by ethanol precipitation and *L. major* minicircle kDNA was detected. *β-catenin* was used as host genomic DNA control. All primer sequences used are listed in Supplementary Table S2.

Supplementary References

1. **Balsalobre A, Damiola F, Schibler U.** 1998. A serum shock induces circadian gene expression in mammalian tissue culture cells. Cell **93**:929-37.
2. **Ramakers C, Ruijter JM, Deprez RH, Moorman AF.** 2003. Assumption-free analysis of quantitative real-time polymerase chain reaction (PCR) data. Neurosci Lett **339**:62-6.
3. **Nicolas L, Prina E, Lang T, Milon G.** 2002. Real-time PCR for detection and quantitation of leishmania in mouse tissues. J Clin Microbiol **40**:1666-9.

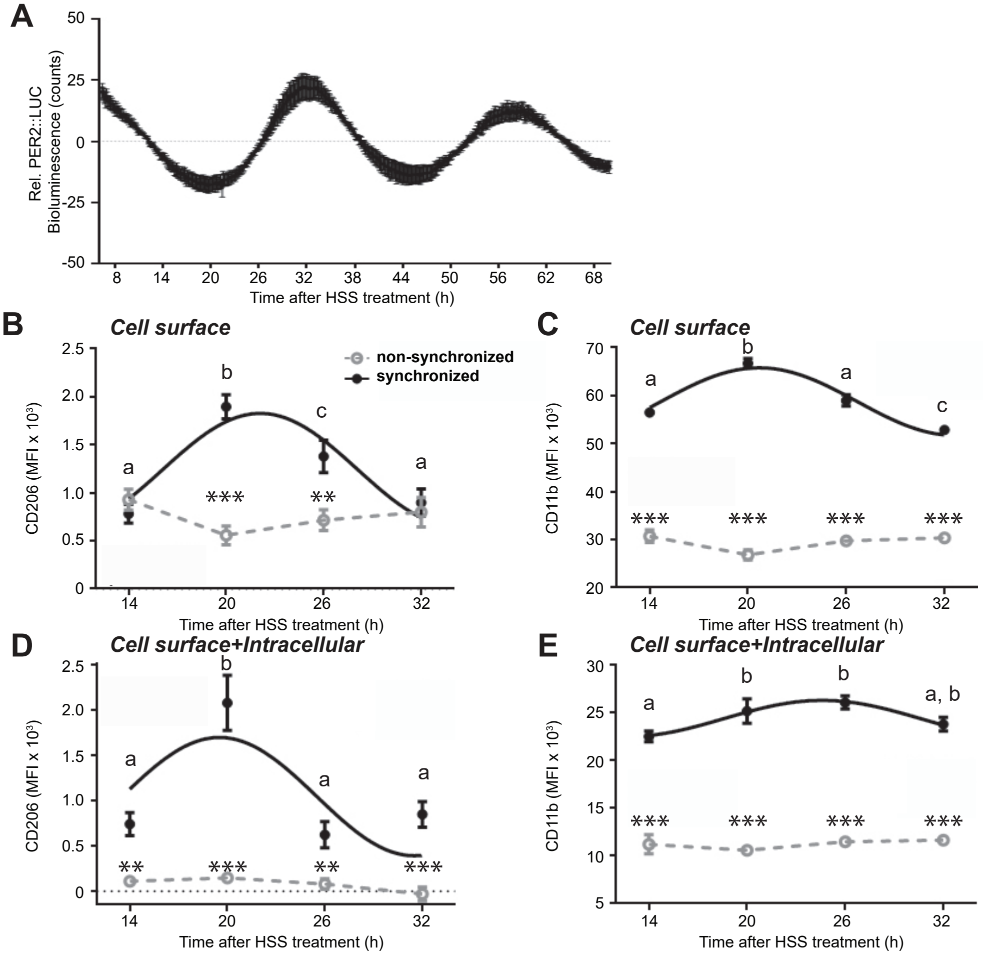

Supplementary Figure S1. Circadian receptor expression after *Leishmania major* infection *in vitro*. (A) Averaged bioluminescence after horse serum shock (HSS) treatment of bone marrow derived macrophages (BMDMs) prepared from PER2::LUC mice, n = 6. Cosine-wave regression, F-test: (B) p < 0.00001. **(B-E)** Mean fluorescence intensity (MFI) of the CD11b and CD206 receptors was quantified by flow cytometry in BMDMs. MFI of cell surface CD206 **(B)** and CD11b **(C)** and intracellular CD206 **(D)** and CD11b **(E)** of non-synchronized BMDMs and of BMDMs 14, 20, 26 and 32 h after a synchronizing HSS. Two-way ANOVA, **(B)** Interaction: F(3, 86) = 13.37, p < 0.0001, n = 11-12, **(C)** Interaction: F(3, 87) = 31.94, p < 0.0001, n = 11-12, **(D)** Interaction: F(3, 68) = 15.59, p < 0.0001, n = 12, **(E)** Interaction: F(3, 86) = 2.832, p = 0.0430, n = 11-12. Cosine-wave regression, F-test: **(B)** p = 0.00001, **(C)** p < 0.00001, **(D)** p = 0.0161, **(E)** p = 0.0213. Significant rhythms are illustrated with fitted cosine curves, otherwise data are simply connected by straight lines between data points, indicating no significant cosine fit. Significance between the two groups are indicated by stars: **p < 0.01; ***p < 0.001. Different letters indicate significant differences between time points within one group (Two-way ANOVA, Tukey's multiple comparisons test; intracellular CD206: n = 6-8, cell surface CD206, cell surface and intracellular CD11b: n = 11-12). Data are presented as mean ± SEM.

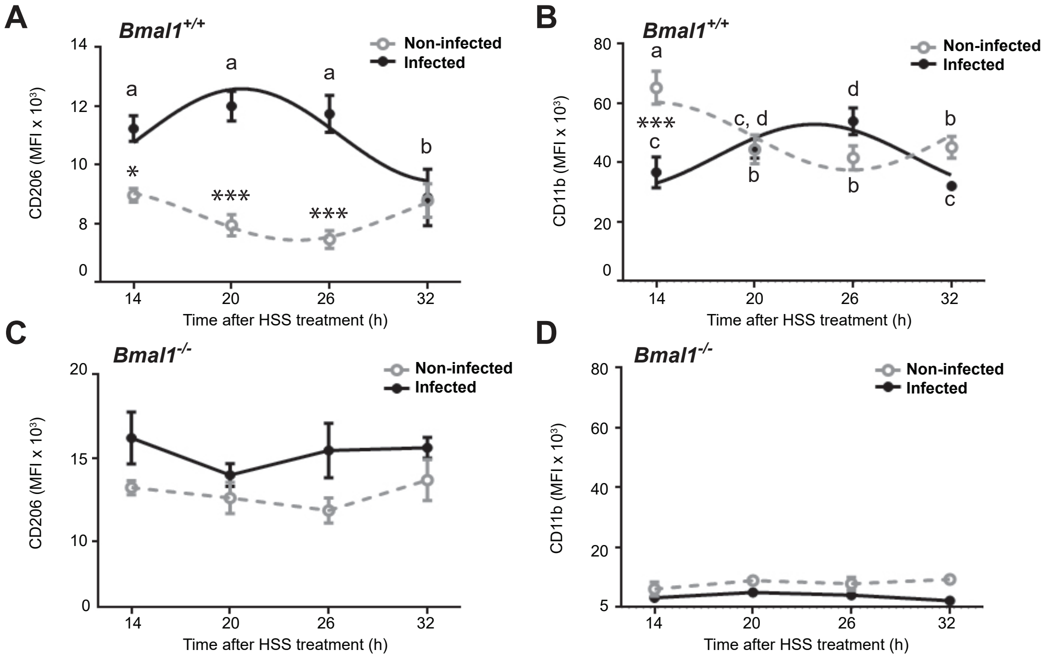

Supplementary Figure S2. Receptor expression is regulated by the macrophage circadian clock *in vitro.* Mean fluorescence intensity (MFI) of the CD206 **(A, C)** and CD11b **(B, D)** receptors was quantified by flow cytometry of bone marrow derived macrophages (BMDMs) obtained from WT (*Bmal1*$^{+/+}$) and *Bmal1*-deficient (*Bmal1*$^{-/-}$) mice, 1 h after infection with *L. major* (infected) or PBS (non-infected) 14, 20, 26 and 32 h after a synchronizing horse serum shock (HSS). Two-way ANOVA, **(A)** Interaction: $F(3, 37) = 6.483$ $p = 0.0012$**,** n = 5-6, **(B)** Interaction: $F(3, 36) = 8.380$, $p = 0.0002$**,** n = 5-6, **(C)** Interaction: $F(3, 36) = 0.4406$, $p = 0.7254$**,** n = 5-6, **(D)** Interaction: $F(3, 29) = 1.351$, $p = 0.2774$**,** n = 3-6. Cosine-wave regression, F-test: (A) non-infected p = 0.050, infected: p = 0.049, **(B)** non-infected p = 0.038, infected: p = 0.018, **(C)** non-infected p = 0.635, infected: p = 0.676, **(D)** non-infected p = 0.968, infected: p = 0.069. Significant rhythms are illustrated with fitted cosine curves, otherwise data are simply connected by straight lines between data points, indicating no significant cosine fit. Significance between the two groups are indicated by stars: *$p < 0.05$; ***$p < 0.001$. Different letters indicate significant differences between time points within one group (Two-way ANOVA, Tukey's multiple comparisons test; *Bmal1*$^{+/+}$: n = 5-6, *Bmal1*$^{-/-}$: n =2-6). Data are presented as mean ± SEM.

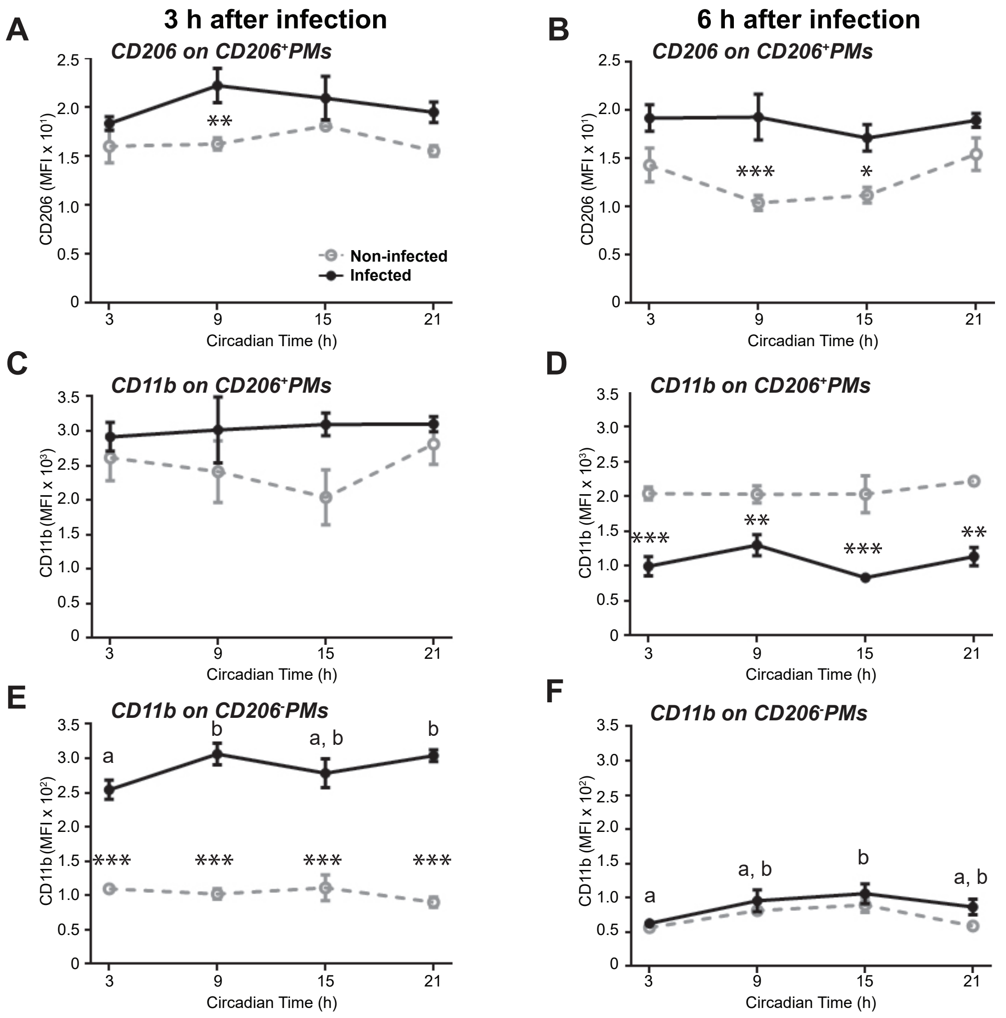

Supplementary Figure S3. Effect of *Leishmania major* infection on receptor expression on macrophages *in vivo*. Mean fluorescence intensity (MFI) of the CD206 **(A, B)** and CD11b **(C-F)** receptors was quantified by flow cytometry on $CD206^+$ or $CD206^-$ macrophages (PMs) in peritoneal exudate cells 3 h **(A, C, E)** and 6 h **(B, D, F)** after injection of PBS or *L. major* at CT3, CT9, CT15 or CT21 in the peritoneal cavity of mice. Two-way ANOVA, **(A)** Group: $F(1, 31) = 18.89$ $p = 0.0001$, n = 4-5, **(B)** Group: $F(3, 32) = 31.49$, $p < 0.0001$, n = 5, **(C)** Group: $F(1, 31) = 6.121$, $p = 0.0190$, n = 4-5, **(D)** Group: $F(1, 32) = 103.6$, $p < 0.0001$, n = 5, **(E)** Group: $F(1, 31) = 387$, $p < 0.0001$, n = 4-5, **(F)** Group: $F(3, 32) = 5.270$, $p = 0.0284$, n = 5. No significant cosine fits. Significance between the two groups are indicated by stars: *$p < 0.05$; **$p < 0.01$; ***$p < 0.001$. Different letters indicate significant differences between time points within one group (Two-way ANOVA, Tukey's multiple comparisons test). Data are presented as mean ± SEM.

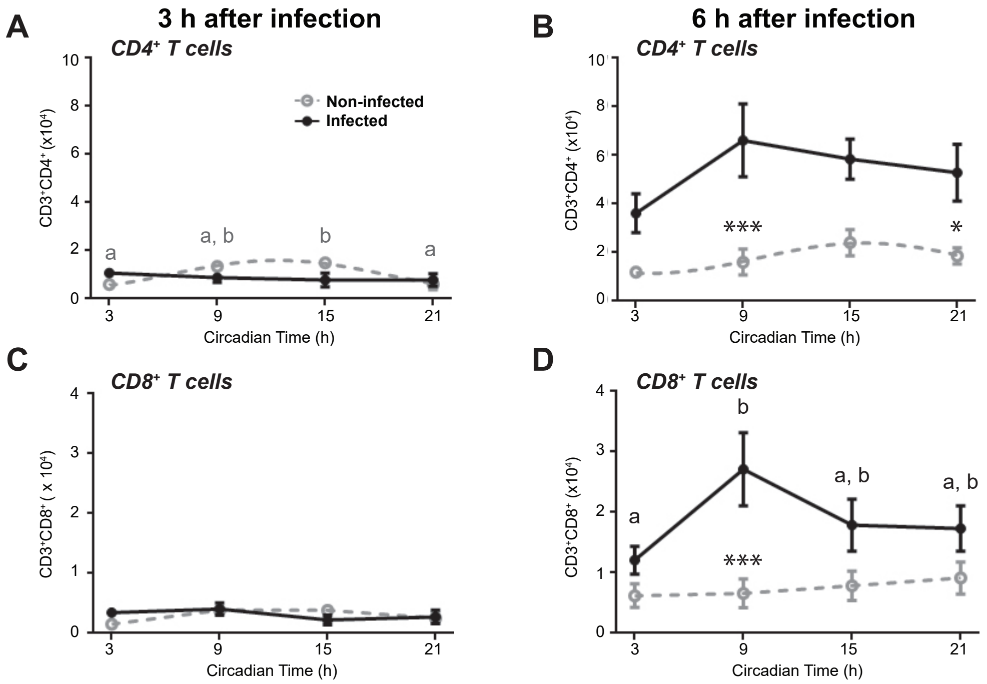

Supplementary Figure S4. T cell recruitment upon *Leishmania major* infection *in vivo.* $CD4^+$ **(A, B)** and $CD8^+$ **(C, D)** T cell numbers in the PECs 3 h **(A, C)** and 6 h **(B, D)** after injection of PBS or *L. major* at CT3, CT9, CT15 or CT21 in the peritoneal cavity of mice. Significant rhythms are illustrated with fitted cosine curves, otherwise data are simply connected by straight lines between data points, indicating no significant cosine fit. Two-way ANOVA, **(A)** Interaction: $F(3, 31) = 3.417$, $p = 0.0294$**,** n = 5-6, **(B)** Interaction: $F(3, 25) = 0.8876$, $p = 0.4611$, n = 3-5, Group: $F(1, 25) = 36.18$, $p < 0.0001$, **(C)** Interaction: $F(3, 32) = 1.773$, $p = 0.1721$, n = 5, **(D)** Interaction: $F(3, 28) = 3.339$, $p = 0.0334$**,** n = 3-5. Cosine-wave regression, F-test: **(A)** non-infected p = 0.006, infected: p = 0.818, **(B)** non-infected p = 0.329, infected: p = 0.437, **(C)** non-infected p = 0.062, infected: p = 0.591, **(D)** non-infected p = 0.843, infected: p = 0.254. Significance between the two groups are indicated by stars: *$p < 0.05$; ***$p < 0.001$. Different letters indicate significant differences between time points within one group (2-way ANOVA, Tukey's multiple comparisons test). Data are presented as mean ± SEM.

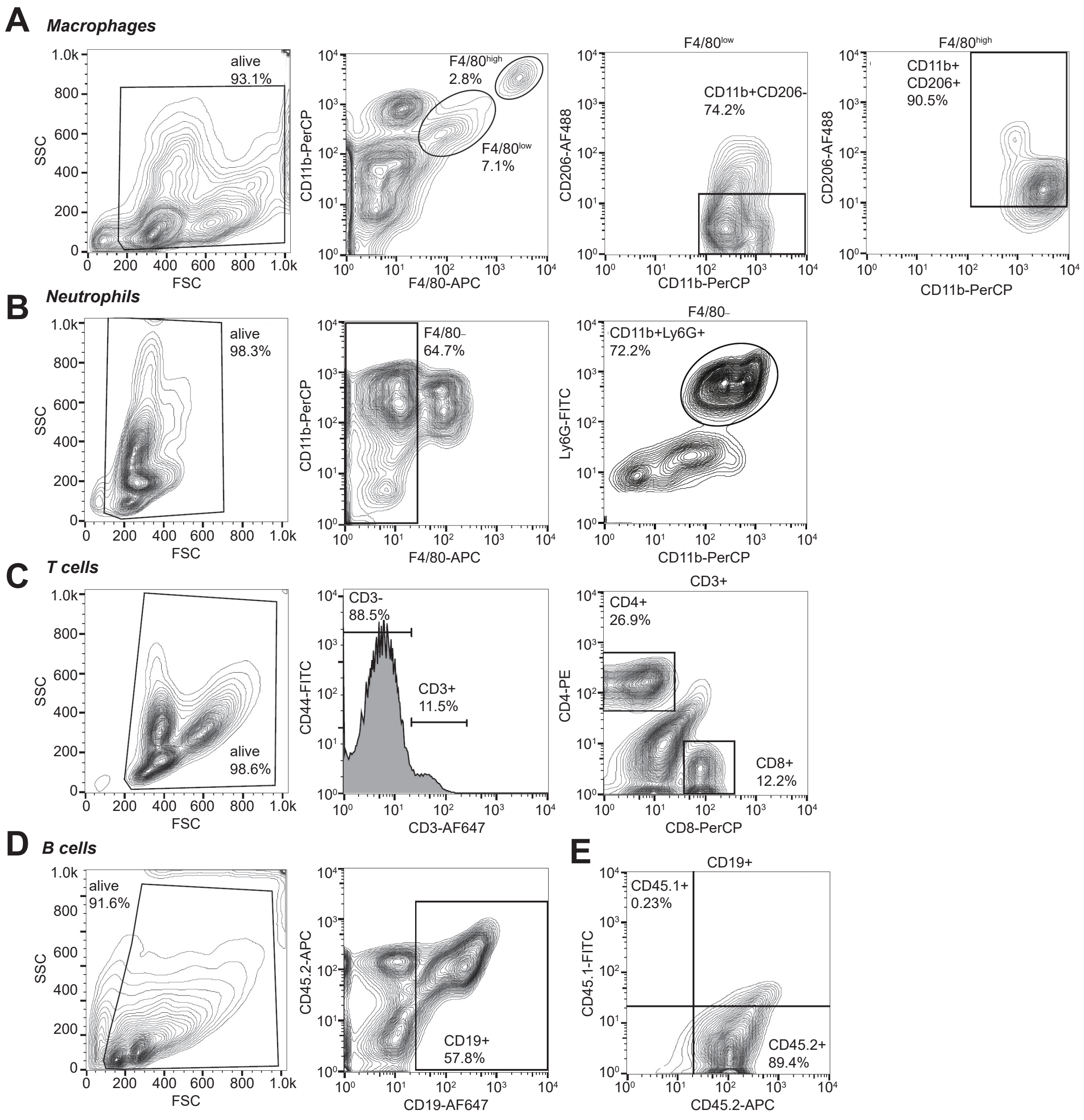

Supplementary Figure S5. Gating strategy for the analysis of immune cell frequencies and receptors in peritoneal exudate cells. (A) Macrophages were assessed based on the alive cell gate, in which F4/80^{low} and F4/80^{high} populations were identified. From these CD11b^{+} and either CD206^{+} or CD206^{-} cells were identified. CD206 and CD11b receptor expression levels were also measured in F4/80^{+}CD11b^{+} macrophages: the MFIs of CD206 was evaluated on CD11b^{+}F4/80^{high} cells, while the MFI of CD11b was examined on CD206^{+}F4/80^{high} and CD206^{-}F4/80^{low} cells. **(B)** Ly6G^{+}CD11b^{+} Neutrophils were gated on F4/80^{-} alive cells. **(C)** CD8^{+} and CD4^{+} T cells were gated on CD3^{+} alive cells. **(D)** B cells were detected as CD19^{+} alive cells. **(E)** B cells are used here as an example of CD45 analysis on immune cells to confirm the efficiency of the bone marrow depletion and graft: CD45.1^{+} and CD45.2^{+} labeling indicates the proportion of cells from the recipient (CD45.1^{+}) or donor (CD45.2^{+}) mouse.

Supplementary Tables

Supplementary Table S1. List of antibodies used

**Antibody**	**Product number**	**Manufacturer**
FITC anti-CD45.1	#110706	Biolegend, San Diego, USA
APC anti-CD45.2	#109814	Biolegend, San Diego, USA
Biotin anti-CD11b	#101204	Biolegend, San Diego, USA
APC anti-F4/80	#123116	Biolegend, San Diego, USA
FITC anti-Ly6G	#127605	Biolegend, San Diego, USA
Alexa Fluor 488 anti-CD206	#141709	Biolegend, San Diego, USA
Alexa Fluor 647 anti-CD3	# 100322	Biolegend, San Diego, USA
PE anti-CD4	#100408	Biolegend, San Diego, USA
PerCP anti-CD8	#100732	Biolegend, San Diego, USA
FITC anti-CD44	#103006	Biolegend, San Diego, USA
Alexa Fluor 647 anti-CD19	#115525	Biolegend, San Diego, USA
PerCP-conjugated streptavidin	#405213	Biolegend, San Diego, USA

Supplementary Table S2. List of primers used

Gene	Forward primer (5'-3')	Reverse primer (5'-3')
Mip-2	ATCCAGAGCTTGAGTGTGACGC	AAGGCAAACTTTTTGACCGCC
Mcp-1	GAAGGAATGGGTCCAGACAT	ACGGGTCAACTTCACATTCA
Mip-1α	GCCACATCGAGGGACTCTTC	GACCAACTGGGAGGGAGATG
Mip-1β	CTCAGCCCTGATGCTTCTCAC	AGAGGGGCAGGAAATCTGAAC
Tnfα	CCCACACCGTCAGCCGATTT	GTCTAAGTACTTGGGCAGATTGACC
Ef1a	TGCCCCAGGACACAGAGACTTCA	AATTCACCAACACCAGCAGCAA
L. major minicircle kDNA	CCTATTTTACACCAACCCCCAGT [JW11]	GGGTAGGGGCGTTCTGCGAAA [JW12]
β-catenin	CTTGGCTGAACCATCAC	GGTCCTCATCGTTTAGCA

Supplementary Table S3. Statistical Information for Figure 1-6

Detailed statistics for Figure 1. *Leishmania major* infection *in vivo* presents a circadian rhythm.

Panel A: Footpad thickness was measured weekly; the difference between the infected and the uninfected paw is shown.

1. **2-way ANOVA**

**p-value (2-way ANOVA)**		
**Inter-action**	**Group**	**Time**
F (27, 134) = 3.907	F (3, 134) = 36.37	F (9, 134) = 132.2
<0.0001	<0.0001	<0.0001

**p-value ((Bonferroni posttest)**							**Sample size**			
**Time (week)**	**CT3 vs. CT9**	**CT3 vs. CT15**	**CT3 vs. CT21**	**CT15 vs CT9**	**CT21 vs CT9**	**CT21 vs CT15**	**CT 3**	**CT 9**	**CT 15**	**CT 21**
**1**	>0.05	>0.05	>0.05	>0.05	>0.05	>0.05	5	4	3	5
**2**	>0.05	>0.05	>0.05	>0.05	>0.05	>0.05	5	4	3	5
**3**	**<0.05**	>0.05	>0.05	>0.05	>0.05	>0.05	5	4	3	5
**4**	**<0.0001**	>0.05	>0.05	**<0.05**	**<0.05**	>0.05	5	4	3	5
**5**	**<0.001**	>0.05	>0.05	**<0.001**	>0.05	>0.05	5	4	3	5
**6**	**<0.001**	>0.05	**<0.001**	>0.05	>0.05	>0.05	5	4	4	5
**7**	**<0.05**	>0.05	**<0.0001**	>0.05	>0.05	>0.05	5	4	3	5
**8**	>0.05	>0.05	**<0.0001**	>0.05	**<0.01**	**<0.01**	5	4	4	5
**9**	>0.05	**<0.05**	**<0.0001**	>0.05	**<0.0001**	**<0.01**	5	4	4	5
**10**	>0.05	>0.05	**<0.0001**	>0.05	**<0.05**	**<0.01**	5	4	4	5

Panel B-D: The parasite load and weight of infected (*L. major*) and non-infected (PBS) draining popliteal lymph nodes was measured 10 weeks post-infection.

1. **Cosine wave regression**

	**p-value (F-test)**	
**Parameter**	***L. major***	**PBS**
**Weight (lymph node)**	0.7274	0.9806
**Parasite load (lymph node)**	**0.0344**	0.9631
**Parasite load (footpad)**	**0.0221**	**0.0439**

2. **2-way ANOVA**

Panel B: Weight (lymph node)

**p-value (2-way ANOVA)**			
**Interaction**	**Group**	**Time**	
0.6411	<0.0001	0.8054	
F (3, 28) = 0.5674	F (1, 28) = 42.94	F (3, 28) = 0.3277	
**p-value (Bonferroni posttest)**		**Sample Size**	
**Circadian Time (h)**	***L. major* vs PBS**	***L. major***	**PBS**
**3**	**<0.001**	5	5
**9**	**<0.05**	4	4
**15**	>0.05	4	4
**21**	**<0.05**	5	5

Panel C: Parasite load (footpad)

**p-value (2-way ANOVA)**			
**Interaction**	**Group**	**Time**	
**0.0186**	**< 0.0001**	**0.0187**	
F (3, 28) = 3.927	F (1, 28) = 41.83	F (3, 28) = 3.917	
**p-value (Bonferroni posttest)**		**Sample Size**	
**Circadian Time (h)**	***L. major* vs PBS**	***L. major***	**PBS**
**3**	>0.05	5	5
**9**	>0.05	4	4
**15**	**<0.001**	4	4
**21**	**<0.0001**	5	5

Panel D: Parasite load (lymph node)

**p-value (2-way ANOVA)**			
**Interaction**	**Group**	**Time**	
**0.0150**	**< 0.0001**	**0.0062**	
F (3, 25) = 4.233	F (1, 25) = 34.09	F (3, 25) = 5.209	
**p-value (Bonferroni posttest)**		**Sample Size**	
**Circadian Time (h)**	***L. major* vs PBS**	***L. major***	**PBS**
**3**	>0.05	5	5
**9**	>0.05	4	4
**15**	**<0.01**	4	4
**21**	**<0.0001**	5	5

Detailed statistics for Figure 2. *Leishmania major* infection is regulated by the circadian clock in macrophages *in vitro*.

Panel A: *L. major-LUC* bioluminescence 1 h after infection (i.e., promastigote attachment) of non-synchronized BMDMs or 20 h/32 h after HSS.

1. **1-way-ANOVA Kruskal-Wallis test**

**p-value**	**0.0209**		
**Circadian Time (h)**	**p-value (Dunn's posttest)**	**Sample Size**	
**20h S vs. NS**	>0.05	5	4
**20h S vs. 32h S**	**<0.05**	5	5
**20h S vs. 32h NS**	>0.05	5	4
**20h NS vs. 32h S**	>0.05	4	5
**20h NS vs. 32h NS**	>0.05	4	4
**32h S vs. 32h NS**	>0.05	5	4

Panel B: *L. major-LUC* bioluminescence 6 h after infection (i.e., promastigote internalization) of non-synchronized BMDMs or 20 h/32 h after HSS.

1. **1-way-ANOVA Kruskal-Wallis test**

**p-value**	**0.0369**		
**Circadian Time (h)**	**p-value (Dunn's posttest)**	**Sample Size**	
**20h S vs. NS**	>0.05	5	4
**20h S vs. 32h S**	**<0.05**	5	5
**20h S vs. 32h NS**	>0.05	5	5
**20h NS vs. 32h S**	>0.05	4	5
**20h NS vs. 32h NS**	>0.05	4	5
**32h S vs. 32h NS**	>0.05	5	5

Panel C: Promastigote attachment to BMDMs of WT or *Bmal1*-deficient mice 1 h after infection 14, 20, 26 or 32 h following a HSS.

1. **Cosine wave regression**

**Genotype**	**p-value (F-test)**
**$Bmall^{+/+}$**	**0.0010**
**$Bmal1^{-/-}$**	0.5361

2. **2-way-ANOVA**

**p-value (2-way ANOVA)**			
**Interaction**	**Group**	**Time**	
**<0.0001**	**0.0003**	**<0.0001**	
F (3, 38) = 16.86	F (1, 38) = 15.96	F (3, 38) = 26.91	
**p-value (Bonferroni posttest)**		**Sample Size**	
**Time (h)**	***L. major* vs PBS**	***L. major***	**PBS**
**14**	**<0.05**	5	6
**20**	**<0.01**	6	6
**26**	**<0.0001**	5	6
**32**	**<0.01**	6	6

**p-value (Bonferroni posttest)**		
**Time (h)**	**$Bmall^{+/+}$**	**$Bmal1^{-/-}$**
**14h vs. 20h**	**<0.0001**	>0.05
**14h vs. 26h**	>0.05	>0.05

**14h vs. 32h**	>0.05	>0.05
**20h vs. 26h**	**<0.0001**	>0.05
**20h vs. 32h**	**<0.0001**	>0.05
**26h vs. 32h**	>0.05	>0.05

Detailed statistics for Figure 3. *Leishmania major* parasite load and immune cell distribution present circadian rhythms *in vivo*.

Panels A, B: The parasite load in *L. major*-infected (and PBS-injected control) mice during the circadian day was determined 3 h or 6 h post-infection by quantifying the *L. major* DNA by quantitative PCR on PEC DNA.

1. **Cosine wave regression**

	**p-value (F-test)**	
**Parameter**	***L. major***	**PBS**
**Parasite load (3h)**	**0.0045**	0.2383
**Parasite load (6h)**	**0.000009**	0.9966

2. **2-way ANOVA (3h after infection)**

**p-value (2-way ANOVA)**			
**Interaction**	**Group**	**Time**	
**0.0004**	**< 0.0001**	**0.0004**	
F (3, 32) = 7.964	F (1, 32) = 49.47	F (3, 32) = 7.964	
**p-value (Bonferroni posttest)**		**Sample Size**	
**Circadian Time (h)**	***L. major* vs PBS**	***L. major***	**PBS**
**3**	>0.05	5	5
**9**	**<0.0001**	5	5
**15**	**<0.001**	5	5
**21**	>0.05	5	5

**p-value (Bonferroni posttest)**		
**Circadian Time (h)**	***L. major***	**PBS**
**CT3 vs. CT9**	**<0.0001**	>0.05
**CT3 vs. CT15**	**<0.01**	>0.05
**CT3 vs. CT21**	>0.05	>0.05
**CT9 vs. CT15**	>0.05	>0.05

**CT9 vs. CT21**	**<0.0001**	>0.05
**CT15 vs. CT21**	**<0.05**	>0.05

3. **2-way ANOVA (6h after infection)**

**p-value (2-way ANOVA)**			
**Interaction**	**Group**	**Time**	
**<0.0001**	**<0.0001**	**<0.0001**	
F (3, 31) = 40.81	F (1, 31) = 238.7	F (3, 31) = 40.86	
**p-value (Bonferroni posttest)**		**Sample Size**	
**Circadian Time (h)**	***L. major* vs PBS**	***L. major***	**PBS**
**3**	>0.05	5	5
**9**	**<0.01**	5	4
**15**	**<0.0001**	5	5
**21**	**<0.0001**	5	5

**p-value (Bonferroni posttest)**		
**Time (h)**	***L. major***	**PBS**
**CT3 vs. CT9**	>0.05	>0.05
**CT3 vs. CT15**	**<0.0001**	>0.05
**CT3 vs. CT21**	**<0.0001**	>0.05
**CT9 vs. CT15**	**<0.0001**	>0.05
**CT9 vs. CT21**	**<0.001**	>0.05
**CT15 vs. CT21**	**<0.0001**	>0.05

Panels C, D: The mouse peritoneal exudate cell (PEC) numbers in *L. major*-infected (and PBS-injected control) mice during the circadian day was determined 3 h or 6 h post-infection by quantifying the *L. major* DNA by quantitative PCR on PEC DNA.

1. **Cosine wave regression**

	**p-value (F-test)**	
**Parameter**	***L. major***	**PBS**
**PEC (3h)**	0.1040	0.1555
**PEC (6h)**	**0.0183**	0.9589

2. **2-way ANOVA (3h after infection)**

**p-value (2-way ANOVA)**			
**Interaction**	**Group**	**Time**	
0.0513	0.6115	**0.0100**	
F (3, 32) = 2.877	F (1, 32) = 0.2632	F (3, 32) = 4.464	
**p-value (Bonferroni posttest)**		**Sample Size**	
**Circadian Time (h)**	***L. major* vs PBS**	***L. major***	**PBS**
**3**	>0.05	5	5
**9**	>0.05	5	5
**15**	>0.05	5	5
**21**	>0.05	5	5

**p-value (Bonferroni posttest)**		
**Circadian Time (h)**	***L. major***	**PBS**
**CT3 vs. CT9**	>0.05	>0.05
**CT3 vs. CT15**	**<0.05**	>0.05
**CT3 vs. CT21**	**<0.05**	>0.05
**CT9 vs. CT15**	>0.05	>0.05
**CT9 vs. CT21**	>0.05	>0.05
**CT15 vs. CT21**	>0.05	>0.05

3. **2-way ANOVA (6h after infection)**

**p-value (2-way ANOVA)**			
**Interaction**	**Group**	**Time**	
**0.0113**	**<0.0001**	**0.0097**	
F (3, 32) = 4.335	F (1, 32) = 397.0	F (3, 32) = 4.487	
**p-value (Bonferroni posttest)**		**Sample Size**	
**Circadian Time (h)**	***L. major* vs PBS**	***L. major***	**PBS**
**3**	**<0.0001**	5	5
**9**	**<0.0001**	5	4
**15**	**<0.0001**	5	5
**21**	**<0.0001**	5	5

p-value (Bonferroni posttest)		
**Time (h)**	***L. major***	**PBS**
**CT3 vs. CT9**	**<0.05**	>0.05
**CT3 vs. CT15**	**<0.0001**	>0.05
**CT3 vs. CT21**	>0.05	>0.05
**CT9 vs. CT15**	>0.05	>0.05
**CT9 vs. CT21**	>0.05	>0.05
**CT15 vs. CT21**	**<0.05**	>0.05

Detailed statistics for Figure 4. Circadian immune cell recruitment after *Leishmania major* infection *in vivo*.

Panel A: Frequencies of recruited F4/80$^-$CD11b$^+$Ly6G$^+$ neutrophils were determined in peritoneal exudate cells (PECs) 3 h **(top)** or 6 h **(bottom)** after injection with PBS or *L. major* in the peritoneal cavity of mice over the circadian day.

1. **Cosine wave regression**

	p-value (F-test)	
**Parameter**	***L. major***	**PBS**
**Neutrophils (3h)**	0.0531	0.4950
**Neutrophils (6h)**	**0.0264**	0.5819

2. **2-way ANOVA (3h after infection)**

p-value (2-way ANOVA)			
**Interaction**	**Group**	**Time**	
**0.0081**	**< 0.0001**	**0.0082**	
F (3, 31) = 4.694	F (1, 31) = 74.98	F (3, 31) = 4.682	
**p-value (Bonferroni posttest)**		**Sample Size**	
**Circadian Time (h)**	***L. major* vs PBS**	***L. major***	**PBS**
**3**	>0.05	5	5
**9**	**< 0.0001**	5	5
**15**	**< 0.001**	5	5
**21**	**< 0.05**	5	5

p-value (Bonferroni posttest)		
**Circadian Time (h)**	***L. major***	**PBS**
**CT3 vs. CT9**	**<0.001**	>0.05

**CT3 vs. CT15**	>0.05	>0.05
**CT3 vs. CT21**	>0.05	>0.05
**CT9 vs. CT15**	**<0.05**	>0.05
**CT9 vs. CT21**	**<0.001**	>0.05
**CT15 vs. CT21**	>0.05	>0.05

3. **2-way ANOVA (6h after infection)**

**p-value (2-way ANOVA)**			
**Interaction**	**Group**	**Time**	
**0.0079**	**<0.0001**	**0.0079**	
F (3, 32) = 4.695	F (1, 32) = 372.0	F (3, 32) = 4.695	
**p-value (Bonferroni posttest)**		**Sample Size**	
**Circadian Time (h)**	***L. major* vs PBS**	***L. major***	**PBS**
**3**	**<0.0001**	5	5
**9**	**<0.0001**	5	4
**15**	**<0.0001**	5	5
**21**	**<0.0001**	5	5

**p-value (Bonferroni posttest)**		
**Time (h)**	***L. major***	**PBS**
**CT3 vs. CT9**	**<0.001**	>0.05
**CT3 vs. CT15**	**<0.01**	>0.05
**CT3 vs. CT21**	>0.05	>0.05
**CT9 vs. CT15**	>0.05	>0.05
**CT9 vs. CT21**	**<0.05**	>0.05
**CT15 vs. CT21**	>0.05	>0.05

Panel B: Mean fluorescence intensity (MFI) of CD11b on neutrophils were determined in peritoneal exudate cells (PECs) 3 h **(top)** or 6 h **(bottom)** after injection with PBS or *L. major* in the peritoneal cavity of mice over the circadian day.

1. **Cosine wave regression**

	**p-value (F-test)**	
**Parameter**	***L. major***	**PBS**
**MFI CD11b (3h)**	**0.0110**	**0.0084**
**MFI CD11b (6h)**	0.7998	0.9185

2. **2-way ANOVA (3h after infection)**

**p-value (2-way ANOVA)**			
**Interaction**	**Group**	**Time**	
**0.0001**	**< 0.0001**	**0.0082**	
F (3, 27) = 10.25	F (1, 27) = 602.8	F (3, 27) = 4.814	
**p-value (Bonferroni posttest)**		**Sample Size**	
**Circadian Time (h)**	***L. major* vs PBS**	***L. major***	**PBS**
**3**	**< 0.0001**	4	4
**9**	**< 0.0001**	5	5
**15**	**< 0.0001**	4	5
**21**	**< 0.0001**	3	5

**p-value (Bonferroni posttest)**		
**Circadian Time (h)**	***L. major***	**PBS**
**CT3 vs. CT9**	**< 0.001**	>0.05
**CT3 vs. CT15**	>0.05	>0.05
**CT3 vs. CT21**	>0.05	>0.05
**CT9 vs. CT15**	>0.05	>0.05
**CT9 vs. CT21**	**< 0.001**	>0.05
**CT15 vs. CT21**	>0.05	>0.05

3. **2-way ANOVA (6h after infection)**

**p-value (2-way ANOVA)**			
**Interaction**	**Group**	**Time**	
0.7334	**<0.0001**	0.6900	
F (3, 31) = 0.4293	F (1, 31) = 23.47	F (3, 31) = 0.4926	
**p-value (Bonferroni posttest)**		**Sample Size**	
**Circadian Time (h)**	***L. major* vs PBS**	***L. major***	**PBS**
**3**	>0.05	5	5
**9**	**<0.05**	5	5
**15**	>0.05	4	5
**21**	**<0.05**	5	5

p-value (Bonferroni posttest)		
**Time (h)**	***L. major***	**PBS**
**CT3 vs. CT9**	>0.05	>0.05
**CT3 vs. CT15**	>0.05	>0.05
**CT3 vs. CT21**	>0.05	>0.05
**CT9 vs. CT15**	>0.05	>0.05
**CT9 vs. CT21**	>0.05	>0.05
**CT15 vs. CT21**	>0.05	>0.05

Panel C: Frequencies of F4/80^{high}CD11b^{+}CD206^{+} peritoneal macrophages (PMs) either 3 h **(top)** or 6 h **(bottom)** after injection of mice with PBS or *L. major* over the circadian day.

1. **Cosine wave regression**

	p-value (F-test)	
**Parameter**	***L. major***	**PBS**
**Peritoneal macrophages (3h)**	0.0544	**0.001**
**Peritoneal macrophages (6h)**	0.9074	0.3363

2. **2-way ANOVA (3h after infection)**

p-value (2-way ANOVA)			
**Interaction**	**Group**	**Time**	
**0.0001**	**< 0.0001**	**0.0004**	
F (3, 30) = 9.992	F (1, 30) = 137.5	F (3, 30) = 8.233	
**p-value (Bonferroni posttest)**		**Sample Size**	
**Circadian Time (h)**	***L. major* vs PBS**	***L. major***	**PBS**
**3**	**< 0.05**	4	5
**9**	**< 0.0001**	5	5
**15**	**< 0.0001**	4	5
**21**	**< 0.01**	5	5

p-value (Bonferroni posttest)		
**Circadian Time (h)**	***L. major***	**PBS**
**CT3 vs. CT9**	>0.05	**< 0.0001**
**CT3 vs. CT15**	>0.05	**< 0.001**
**CT3 vs. CT21**	>0.05	>0.05
**CT9 vs. CT15**	>0.05	>0.05
**CT9 vs. CT21**	>0.05	**< 0.0001**
**CT15 vs. CT21**	>0.05	**< 0.01**

3. **2-way ANOVA (6h after infection)**

**p-value (2-way ANOVA)**			
**Interaction**	**Group**	**Time**	
0.3995	**< 0.0001**	0.2448	
F (3, 31) = 1.015	F (1, 31) = 24.55	F (3, 31) = 1.459	
**p-value (Bonferroni posttest)**		**Sample Size**	
**Circadian Time (h)**	***L. major* vs PBS**	***L. major***	**PBS**
**3**	>0.05	5	4
**9**	>0.05	5	5
**15**	**<0.01**	5	5
**21**	>0.05	5	5

**p-value (Bonferroni posttest)**		
**Time (h)**	***L. major***	**PBS**
**CT3 vs. CT9**	>0.05	>0.05
**CT3 vs. CT15**	>0.05	>0.05
**CT3 vs. CT21**	>0.05	>0.05
**CT9 vs. CT15**	>0.05	>0.05
**CT9 vs. CT21**	>0.05	>0.05
**CT15 vs. CT21**	>0.05	>0.05

Panel D: Frequencies of F4/80^{low}CD11b^{+}CD206^{-} PMs either 3 h **(top)** or 6 h **(bottom)** after injection of mice with PBS or *L. major* over the circadian day.

1. **Cosine wave regression**

	**p-value (F-test)**	
**Parameter**	***L. major***	**PBS**
**CD206^{-} PMs (3h)**	0.1503	**0.0211**
**CD206^{-} PMs (6h)**	**0.0087**	0.0554

2. **2-way ANOVA (3h after infection)**

**p-value (2-way ANOVA)**		
**Interaction**	**Group**	**Time**
0.3629	**< 0.0001**	**0.0003**
F (3, 32) = 1.101	F (1, 32) = 19.75	F (3, 32) = 8.277

**p-value (Bonferroni posttest)**		**Sample Size**	
**Circadian Time (h)**	***L. major* vs PBS**	***L. major***	**PBS**
**3**	> 0.05	5	5
**9**	**< 0.05**	5	5
**15**	> 0.05	5	5
**21**	**< 0.05**	5	5

**p-value (Bonferroni posttest)**		
**Circadian Time (h)**	***L. major***	**PBS**
**CT3 vs. CT9**	>0.05	**< 0.05**
**CT3 vs. CT15**	>0.05	**< 0.01**
**CT3 vs. CT21**	>0.05	**< 0.01**
**CT9 vs. CT15**	>0.05	>0.05
**CT9 vs. CT21**	>0.05	>0.05
**CT15 vs. CT21**	>0.05	>0.05

3. **2-way ANOVA (6h after infection)**

**p-value (2-way ANOVA)**			
**Interaction**	**Group**	**Time**	
**0.0023**	**< 0.0001**	**0.0008**	
F (3, 32) = 6.005	F (1, 32) = 401.7	F (3, 32) = 7.175	
**p-value (Bonferroni posttest)**		**Sample Size**	
**Circadian Time (h)**	***L. major* vs PBS**	***L. major***	**PBS**
**3**	**< 0.0001**	5	5
**9**	**< 0.0001**	5	5
**15**	**< 0.0001**	5	5
**21**	**< 0.0001**	5	5

**p-value (Bonferroni posttest)**		
**Time (h)**	***L. major***	**PBS**
**CT3 vs. CT9**	>0.05	>0.05
**CT3 vs. CT15**	**< 0.0001**	>0.05
**CT3 vs. CT21**	>0.05	>0.05
**CT9 vs. CT15**	**< 0.0001**	>0.05
**CT9 vs. CT21**	>0.05	>0.05
**CT15 vs. CT21**	**< 0.01**	>0.05

Detailed statistics for Figure 5. Parasite load and immune cell recruitment after *Leishmania major* infection is controlled by the circadian clock in phagocytic immune cells *in vivo*.

1. **2-way ANOVA**

**p-value (2-way ANOVA)**			
**Parameter**	**Interaction**	**Group**	**Time**
**Parasite load**	**0.0023**	**< 0.0001**	**0.0008**
	$F(3, 32) = 6.005$	$F(1, 32) = 401.7$	$F(3, 32) = 7.175$
**PECs**	**0.0056**	**0.0909**	**< 0.0001**
	$F(3, 20) = 5.668$	$F(1, 20) = 3.156$	$F(3, 20) = 21.01$
**Neutrophils**	**0.0147**	**< 0.0001**	**0.0194**
	$F(3, 17) = 4.674$	$F(3, 17) = 117.7$	$F(1, 17) = 6.668$
**CD206^{+} PMs**	**0.0031**	**< 0.0001**	0.0830
	$F(3, 16) = 7.072$	$F(3, 16) = 22.93$	$F(1, 16) = 3.419$
**CD206^{-} PMs**	**0.0014**	**< 0.0001**	**0.0138**
	$F(3, 17) = 8.138$	$F(3, 17) = 66.52$	$F(1, 17) = 7.539$
**CD11b on 206^{-} PMs**	**0.0413**	**0.0012**	**0.0050**
	$F(3, 17) = 3.416$	$F(3, 17) = 8.354$	$F(1, 17) = 10.40$
**CD11b on 206^{+} PMs**	0.7131	**< 0.0001**	0.7082
	$F(3, 19) = 0.4606$	$F(3, 19) = 23.26$	$F(1, 19) = 0.1444$
**CD206 on 206^{+} PMs**	0.1988	**< 0.0001**	**0.0451**
	$F(3, 18) = 1.719$	$F(3, 18) = 15.86$	$F(1, 18) = 4.636$
**CD11b on Neutrophils**	0.6984	**0.0002**	0.6180
	$F(3, 19) = 0.4824$	$F(3, 19) = 11.05$	$F(1, 19) = 0.2571$

**p-value (Bonferroni posttest): CT3 vs. CT15**				
	**WT-BM**		**KO-BM**	
**Parameter**	***L. major***	**PBS**	***L. major***	**PBS**
**Parasite load**	**< 0.001**	>0.05	>0.05	>0.05
**PECs**	**< 0.001**	>0.05	>0.05	>0.05
**Neutrophils**	**< 0.001**	>0.05	>0.05	>0.05
**CD206^{+} PMs**	>0.05	**< 0.001**	>0.05	>0.05
**CD206^{-} PMs**	**< 0.0001**	>0.05	>0.05	>0.05

**CD11b on 206^- PMs**	>0.05	**< 0.01**	>0.05	>0.05
**CD11b on 206^+ PMs**	>0.05	>0.05	>0.05	>0.05
**CD206 on 206^+ PMs**	**< 0.01**	>0.05	>0.05	>0.05
**CD11b on Neutrophils**	>0.05	>0.05	>0.05	>0.05
**Sample size**	4-6	3-4	3-4	2

Detailed statistics for Figure 6. Circadian expression of chemoattractants after *L. major* infection *in vivo*.

Panel A: *Mip2, Mcp1, Mip1α, Mip1β* and *Tnfα* expression in peritoneal exudate cells (PECs) of *L. major*-infected or PBS-injected mice 3 h after injection.

1. **Cosine wave regression**

	**p-value (F-test)**	
**Cytokine/Chemokine**	***L. major***	**PBS**
Mip2	0.0663	0.3125
Mcp1	**0.0046**	0.7459
Mip1α	0.1189	0.1781
Mip1β	**0.0169**	0.4793
Tnfα	**0.0235**	0.8177

2. **2-way ANOVA (*Mip2*)**

**p-value (2-way ANOVA)**		
**Interaction**	**Group**	**Time**
**0.0497**	**< 0.0001**	**0.0478**
F (3, 32) = 2.907	F (1, 32) = 70.26	F (3, 32) = 2.943

**p-value (Bonferroni posttest)**		**Sample Size**	
**Circadian Time (h)**	***L. major* vs PBS**	***L. major***	**PBS**
**3**	> 0.05	5	5
**9**	**< 0.001**	5	5
**15**	**< 0.0001**	5	5
**21**	**< 0.001**	5	5

**p-value (Bonferroni posttest)**		
**Circadian Time (h)**	***L. major***	**PBS**
**CT3 vs. CT9**	>0.05	>0.05
**CT3 vs. CT15**	**<0.01**	>0.05
**CT3 vs. CT21**	>0.05	>0.05

**CT9 vs. CT15**	>0.05	>0.05
**CT9 vs. CT21**	>0.05	>0.05
**CT15 vs. CT21**	>0.05	>0.05

3. **2-way ANOVA (*Mcp1*)**

**p-value (2-way ANOVA)**			
**Interaction**	**Group**	**Time**	
0.0622	**< 0.0001**	**0.0216**	
F (3, 24) = 2.790	F (1, 24) = 22.26	F (3, 24) = 3.877	
**p-value (Bonferroni posttest)**		**Sample Size**	
**Circadian Time (h)**	***L. major* vs PBS**	***L. major***	**PBS**
**3**	> 0.05	5	2
**9**	**< 0.01**	5	2
**15**	**< 0.05**	5	4
**21**	> 0.05	5	5

**p-value (Bonferroni posttest)**		
**Circadian Time (h)**	***L. major***	**PBS**
**CT3 vs. CT9**	**<0.05**	>0.05
**CT3 vs. CT15**	>0.05	>0.05
**CT3 vs. CT21**	>0.05	>0.05
**CT9 vs. CT15**	>0.05	>0.05
**CT9 vs. CT21**	**<0.001**	>0.05
**CT15 vs. CT21**	**<0.05**	>0.05

4. **2-way ANOVA (*Mip1α*)**

**p-value (2-way ANOVA)**			
**Interaction**	**Group**	**Time**	
0.1044	**< 0.0001**	**0.0442**	
F (3, 31) = 2.230	F (1, 31) = 49.19	F (3, 31) = 3.02	
**p-value (Bonferroni posttest)**		**Sample Size**	
**Circadian Time (h)**	***L. major* vs PBS**	***L. major***	**PBS**
**3**	> 0.05	5	5
**9**	**< 0.01**	5	4
**15**	**< 0.0001**	5	5

**21**	> 0.05	5	5

**p-value (Bonferroni posttest)**		
**Circadian Time (h)**	***L. major***	**PBS**
**CT3 vs. CT9**	**<0.05**	>0.05
**CT3 vs. CT15**	>0.05	>0.05
**CT3 vs. CT21**	>0.05	>0.05
**CT9 vs. CT15**	>0.05	>0.05
**CT9 vs. CT21**	>0.05	>0.05
**CT15 vs. CT21**	**<0.01**	>0.05

5. **2-way ANOVA (*Mip1β*)**

**p-value (2-way ANOVA)**			
**Interaction**	**Group**	**Time**	
**0.0205**	**0.0005**	**0.0198**	
F (3, 24) = 3.930	F (1, 24) = 15.92	F (3, 24) = 3.970	
**p-value (Bonferroni posttest)**		**Sample Size**	
**Circadian Time (h)**	***L. major* vs PBS**	***L. major***	**PBS**
**3**	> 0.05	5	5
**9**	**< 0.01**	5	4
**15**	**< 0.0001**	5	5
**21**	> 0.05	5	5

**p-value (Bonferroni posttest)**		
**Circadian Time (h)**	***L. major***	**PBS**
**CT3 vs. CT9**	>0.05	>0.05
**CT3 vs. CT15**	**<0.001**	>0.05
**CT3 vs. CT21**	>0.05	>0.05
**CT9 vs. CT15**	**<0.05**	>0.05
**CT9 vs. CT21**	>0.05	>0.05
**CT15 vs. CT21**	**<0.0001**	>0.05

6. **2-way ANOVA (*Tnfα*)**

**p-value (2-way ANOVA)**		
**Interaction**	**Group**	**Time**
**0.0229**	**<0.0001**	**0.0117**
F (3, 28) = 3.714	F (1, 28) = 63.65	F (3, 28) = 4.404
**p-value (Bonferroni**		**Sample Size**

posttest)			
**Circadian Time (h)**	***L. major* vs PBS**	***L. major***	**PBS**
**3**	> 0.05	5	4
**9**	**< 0.01**	5	4
**15**	**< 0.0001**	5	4
**21**	> 0.05	5	4

**p-value (Bonferroni posttest)**		
**Circadian Time (h)**	***L. major***	**PBS**
**CT3 vs. CT9**	>0.05	>0.05
**CT3 vs. CT15**	**<0.001**	>0.05
**CT3 vs. CT21**	>0.05	>0.05
**CT9 vs. CT15**	>0.05	>0.05
**CT9 vs. CT21**	>0.05	>0.05
**CT15 vs. CT21**	**<0.001**	>0.05

Panel B: *Mip2, Mcp1, Mip1α, Mip1β* and *Tnfα* expression in peritoneal exudate cells (PECs) of *L. major*-infected or PBS-injected mice 6 h after injection.

1. **Cosine wave regression**

	**p-value (F-test)**	
**Cytokine/Chemokine**	***L. major***	**PBS**
Mip2	**0.0003**	0.1120
Mcp1	**0.0017**	0.0683
Mip1α	**0.0500**	0.1135
Mip1β	**0.0024**	0.4121
Tnfα	0.0886	0.7824

2. **2-way ANOVA (*Mip2*)**

**p-value (2-way ANOVA)**			
**Interaction**	**Group**	**Time**	
**< 0.0001**	**< 0.0001**	**< 0.0001**	
F (3, 32) = 18.50	F (1, 32) = 128.5	F (3, 32) = 18.46	
**p-value (Bonferroni posttest)**		**Sample Size**	
**Circadian Time (h)**	***L. major* vs PBS**	***L. major***	**PBS**
**3**	> 0.05	5	5
**9**	**<0.0001**	5	5
**15**	**< 0.0001**	5	5
**21**	**< 0.01**	5	5

p-value (Bonferroni posttest)		
**Circadian Time (h)**	***L. major***	**PBS**
**CT3 vs. CT9**	**<0.05**	>0.05
**CT3 vs. CT15**	**<0.0001**	>0.05
**CT3 vs. CT21**	>0.05	>0.05
**CT9 vs. CT15**	**<0.0001**	>0.05
**CT9 vs. CT21**	>0.05	>0.05
**CT15 vs. CT21**	**<0.0001**	>0.05

3. **2-way ANOVA (*Mcp1*)**

**p-value (2-way ANOVA)**			
**Interaction**	**Group**	**Time**	
**< 0.0001**	**< 0.0001**	**< 0.0001**	
F (3, 32) = 30.01	F (1, 32) = 455.8	F (3, 32) = 30.65	
**p-value (Bonferroni posttest)**		**Sample Size**	
**Circadian Time (h)**	***L. major* vs PBS**	***L. major***	**PBS**
**3**	**<0.0001**	5	5
**9**	**<0.0001**	5	5
**15**	**<0.0001**	5	5
**21**	**<0.0001**	5	5

**p-value (Bonferroni posttest)**		
**Circadian Time (h)**	***L. major***	**PBS**
**CT3 vs. CT9**	**<0.0001**	>0.05
**CT3 vs. CT15**	>0.05	>0.05
**CT3 vs. CT21**	>0.05	>0.05
**CT9 vs. CT15**	**<0.0001**	>0.05
**CT9 vs. CT21**	**<0.0001**	>0.05
**CT15 vs. CT21**	>0.05	>0.05

4. **2-way ANOVA (*Mip1α*)**

**p-value (2-way ANOVA)**		
**Interaction**	**Group**	**Time**
**0.0021**	**< 0.0001**	**0.0023**
F (3, 32) = 6.094	F (1, 32) = 145.8	F (3, 32) = 6.012
**p-value (Bonferroni posttest)**		**Sample Size**

Circadian Time (h)	*L. major* vs PBS	*L. major*	PBS
**3**	**<0.0001**	5	5
**9**	**< 0.01**	5	4
**15**	**< 0.0001**	5	5
**21**	**<0.0001**	5	5

**p-value (Bonferroni posttest)**		
**Circadian Time (h)**	***L. major***	**PBS**
**CT3 vs. CT9**	>0.05	>0.05
**CT3 vs. CT15**	**<0.01**	>0.05
**CT3 vs. CT21**	>0.05	>0.05
**CT9 vs. CT15**	**<0.0001**	>0.05
**CT9 vs. CT21**	**<0.05**	>0.05
**CT15 vs. CT21**	**<0.05**	>0.05

5. 2-way ANOVA (*Mip1β*)

**p-value (2-way ANOVA)**			
**Interaction**	**Group**	**Time**	
**<0.0001**	**<0.0001**	**<0.0001**	
F (3, 32) = 14.23	F (1, 32) = 163.5	F (3, 32) = 14.22	
**p-value (Bonferroni posttest)**		**Sample Size**	
**Circadian Time (h)**	***L. major* vs PBS**	***L. major***	**PBS**
**3**	**< 0.001**	5	5
**9**	**< 0.05**	5	5
**15**	**< 0.0001**	5	5
**21**	**< 0.0001**	5	5

**p-value (Bonferroni posttest)**		
**Circadian Time (h)**	***L. major***	**PBS**
**CT3 vs. CT9**	>0.05	>0.05
**CT3 vs. CT15**	**<0.0001**	>0.05
**CT3 vs. CT21**	>0.05	>0.05
**CT9 vs. CT15**	**<0.0001**	>0.05
**CT9 vs. CT21**	**<0.01**	>0.05
**CT15 vs. CT21**	**<0.001**	>0.05

6. **2-way ANOVA (*Tnfα*)**

**p-value (2-way ANOVA)**			
**Interaction**	**Group**	**Time**	
**0.0443**	**<0.0001**	**0.0488**	
F (3, 32) = 3.015	F (1, 32) = 164.2	F (3, 32) = 2.923	
**p-value (Bonferroni posttest)**		**Sample Size**	
**Circadian Time (h)**	***L. major* vs PBS**	***L. major***	**PBS**
**3**	**< 0.0001**	5	5
**9**	**< 0.001**	5	5
**15**	**< 0.0001**	5	5
**21**	**< 0.0001**	5	5

**p-value (Bonferroni posttest)**		
**Circadian Time (h)**	***L. major***	**PBS**
**CT3 vs. CT9**	>0.05	>0.05
**CT3 vs. CT15**	**<0.05**	>0.05
**CT3 vs. CT21**	>0.05	>0.05
**CT9 vs. CT15**	**< 0.01**	>0.05
**CT9 vs. CT21**	>0.05	>0.05
**CT15 vs. CT21**	>0.05	>0.05

Panel C: Cytokine/chemokine expression 6 h after *L. major* or PBS injection in chimeric mice consisting of irradiated B6.SJL hosts that have received bone marrow cells from either *Bmal1*$^{+/+}$ OT-1 *Rag2*$^{-/-}$ (WT-BM) or *Bmal1*$^{-/-}$ OT-1 *Rag2*$^{-/-}$ donors (KO-BM).

1. **2-way ANOVA**

**p-value (2-way ANOVA)**			
**Cytokine / Chemokine**	**Interaction**	**Group**	**Time**
Mip2	**0.0366**	**< 0.0001**	0.1209
	F (3, 23) = 3.347	F (3, 23) = 22.12	F (1, 23) = 2.594
Mcp1	0.2200	**< 0.0001**	0.4765
	F (3, 21) = 1.597	F (3, 21) = 50.17	F (1, 21) = 0.5254
Mip1α	**0.0296**	**< 0.0001**	0.0645
	F (3, 22) = 3.601	F (3, 22) = 30.53	F (1, 22) = 3.788

Mip1β	**0.0082**	**< 0.0001**	0.1785
	F (3, 21) = 5.112	F (3, 21) = 31.55	F (1, 21) = 1.938
Tnfα	0.2385	**< 0.0001**	0.1690
	F (3, 21) = 1.520	F (3, 21) = 17.13	F (1, 21) = 2.029

**p-value (Bonferroni posttest): CT3 vs. CT15**				
	**WT-BM**		**KO-BM**	
**Cytokine / Chemokine**	***L. major***	**PBS**	***L. major***	**PBS**
Mip2	**< 0.01**	>0.05	>0.05	>0.05
Mcp1	>0.05	>0.05	>0.05	>0.05
Mip1α	**< 0.01**	>0.05	>0.05	>0.05
Mip1β	**< 0.01**	>0.05	>0.05	>0.05
Tnfα	>0.05	>0.05	>0.05	>0.05
**Sample size**	6	4	3-4	2
